# A multi-fidelity poroelastic finite element and machine learning framework for characterizing respiratory mechanics in porcine lungs

**DOI:** 10.1007/s10237-026-02100-7

**Published:** 2026-07-01

**Authors:** Edwin E. Aigbokhan, Olusola A. Olabanjo, Emmanuel A. Akor, David W. Kaczka, Mingchao Cai

**Affiliations:** 1 Department of Mathematics, Morgan State University, Baltimore, MD USA; 2 Center for Equitable AI and Machine Learning Systems, Morgan State University, Baltimore, MD USA; 3 Roy J. Carver Department of Biomedical Engineering, University of Iowa, Iowa City, IA USA; 4 Department of Anesthesia, University of Iowa, Iowa City, IA USA; 5 Department of Radiology, University of Iowa, Iowa City, IA USA

**Keywords:** Poroelasticity, Finite-element modeling, Multi-fidelity Gaussian process, Sobol sensitivity analysis, Uncertainty quantification, Lung biomechanics, Parameter identification

## Abstract

Accurate and rapid characterization of lung mechanics remains a central challenge in respiratory disease management. Physics-informed poroelastic finite-element (FE) models resolve detailed tissue–airflow interactions but are computationally prohibitive for real-time or large-scale clinical applications, while lumped-parameter models sacrifice mechanistic fidelity for efficiency. In this work, we present a porcine-specific, multi-fidelity computational framework that integrates poroelastic FE modeling with machine learning to enable rapid, uncertainty-aware estimation of respiratory compliance (${ C}_{\textrm{rs}}$) and resistance (${ R}_{\textrm{rs}}$). High- and low-fidelity simulations are generated from CT-derived porcine lung geometries by sampling a physiologically relevant parameter space, and the resulting pressure–volume dynamics are used in an inverse modeling procedure to infer global respiratory mechanics. A key result is that multi-fidelity Gaussian process (MF-GP) surrogates achieve accurate predictions of ${ C}_{\textrm{rs}}$ and ${ R}_{\textrm{rs}}$ with errors below 5% relative to high-fidelity simulations, while providing computational speedups of over five orders of magnitude. In contrast, neural network (NN) surrogates exhibit relatively poor generalization in the data-scarce regime considered, highlighting the importance of model selection for scientific machine learning under limited high-fidelity data availability. Beyond predictive performance, global sensitivity analysis reveals a clear mechanistic separation in parameter influence: compliance is primarily governed by elastic stiffness and chest-wall coupling, whereas resistance is dominated by permeability. The weak interaction effects observed support an approximately additive response structure, enabling robust parameter identifiability and reduced-order representations of the inverse problem. The framework is validated against independent ventilator measurements from porcine lungs, showing strong agreement within clinically observed ranges. Overall, this study provides new insight into the structure of the inverse problem in poroelastic lung modeling and establishes a computationally efficient pathway for uncertainty-aware prediction and parameter estimation, with potential applications in personalized ventilation and preclinical study design.

## Introduction

Lung disease research has gained increasing awareness in light of the devastating global impact of respiratory illnesses, most notably the COVID-19 pandemic, which has resulted in substantial mortality and long-term pulmonary impairment (Levine and Marciniuk 2022; Neelakantan et al. 2022). Beyond COVID-19, the rising incidence of chronic obstructive pulmonary disease (COPD), acute respiratory distress syndrome (ARDS), asthma, and pulmonary fibrosis continues to impose significant clinical and socioeconomic burdens, further exacerbated by aging populations and environmental stressors (Hasan et al. 2020; Wu et al. 2020; Wang et al. 2022; Shahid et al. 2024; Lu et al. 2025).

Accurately modeling and quantifying lung disease, however, remains fundamentally challenging. Diseased lungs exhibit pronounced nonlinearity, spatial heterogeneity, and dynamic instability, such that conventional global descriptors derived from ventilator waveform data provide only coarse representations of true physiological behavior. These limitations are compounded by measurement noise, spontaneous breathing efforts, airway instrumentation, and time-dependent phenomena such as alveolar recruitment and derecruitment. As a result, a significant disconnect persists between the complexity of pulmonary pathophysiology and the simplified models commonly used in clinical practice (Bates and Ht 2009; Kaczka and Dellacá 2011). In clinical settings, lung mechanics are most commonly characterized using the single-compartment model, which represents the respiratory system as a linear resistance–compliance network. This model is widely adopted due to its simplicity and ability to provide rapid bedside estimates of global lung mechanics (Bates and Ht 2009). The airway pressure is expressed as

1 $$\begin{aligned} P(t) = \frac{V(t)}{{ C}_{\text {rs}}} + { R}_{\text {rs}}\,\dot{V}(t) + P_0, \end{aligned}$$

where *V*(*t*) is lung volume, $\dot{V}(t)$ is flow rate, ${ C}_{\text {rs}}$ and ${ R}_{\text {rs}}$ denote respiratory compliance and resistance, respectively, and $P_0$ is the positive end-expiratory pressure (PEEP). Despite its practicality, reliable estimation of ${ C}_{\text {rs}}$ and ${ R}_{\text {rs}}$ is difficult in real clinical environments due to limited data quality and non-ideal acquisition conditions, thereby limiting its utility for precision ventilation and patient-specific therapy.

Recent advances in poroelastic and poro-hyperelastic lung modeling have significantly improved the physiological realism of continuum simulations. Badrou et al. (Badrou et al. 2025) developed a poro-hyperelastic finite-element (FE) framework validated against experimental pressure, volume, and strain data, while Manoochehrtayebi et al. (Manoochehrtayebi et al. 2026) introduced a microscale poromechanical model incorporating alveolar mechanics and surface tension effects. Comprehensive reviews (Neelakantan et al. 2022) further highlight rapid progress in multi-scale and whole-lung modeling. In parallel, physics-based poroelastic FE formulations (Berger et al. 2016; Birzle et al. 2019; Avilés-Rojas and Hurtado 2022; Olabanjo et al. 2025) have enabled anatomically realistic simulations of lung deformation and fluid–solid coupling. Nevertheless, these approaches remain computationally intensive and are not designed for rapid parameter estimation or uncertainty quantification.

Further improvements in physical fidelity have been achieved through organ-scale poromechanical models. Patte et al. (Patte et al. 2022b) proposed a quasi-static formulation capturing equilibrium lung deformation, while Peyraut and Genet (Peyraut and Genet 2024) incorporated gravity and heterogeneous pleural pressure distributions to better represent regional ventilation heterogeneity. Although these models provide detailed forward simulations, they do not directly address efficient parameter inference or real-time predictive capability. Complementary efforts have advanced inverse modeling and uncertainty quantification (UQ) for personalized lung mechanics.  Patte et al. (2022a) estimated regional pulmonary compliance using image-based poromechanical models, and  Laville et al. (2023) investigated parameter identifiability and sensitivity to optimization formulations. More recently,  Peyraut and Genet (2025) introduced a probabilistic inverse UQ framework for pulmonary digital twins. However, these approaches typically rely on repeated high-fidelity simulations, limiting their scalability for large-scale or real-time applications.

While substantial progress has been achieved in human lung modeling (Gerard et al. 2020; Barahona et al. 2024), Biot-based poroelastic formulations have also been applied to porcine lungs (Henry and Royston 2018; Peng et al. 2016; Dai et al. 2014b, a), primarily in the context of acoustical and vibrational mechanics at high frequencies (20–2000 Hz) under small-amplitude perturbations. In contrast, respiratory mechanics relevant to ventilation and ventilator-induced lung injury (VILI) operate in a quasi-static, low-frequency regime involving large deformations, which remains insufficiently explored in porcine-specific modeling. This gap is particularly significant because porcine lungs constitute the primary large-animal model for studying VILI and optimizing ventilation strategies (Yoshida et al. 2018; Giffin et al. 2025; Ruiz 2025). Direct translation of human-based models is limited by species-specific differences in lung geometry, airway architecture, tissue mechanics, and chest-wall interactions (Judge et al. 2014). At the same time, porcine lungs exhibit strong physiological similarity to human lungs in airway branching, organ size, and alveolar organization, while enabling controlled experimental measurements under well-defined conditions (Suki et al. 2005; Suki and Bates 2025). Although porcine lung tissue is moderately stiffer, key functional characteristics (e.g., compliance, resistance, nonlinear pressure–volume behavior, as well as viscoelastic dynamics central to VILI)—remain comparable across species depending on lung size (Kaczka and Dellacá 2011; Bates and Ht 2009; Suki and Bates 2025). Consequently, porcine modeling provides a critical bridge between idealized human simulations and experimentally validated, clinically relevant lung mechanics.

To address this gap, we develop a porcine-specific multi-fidelity poroelastic framework that integrates CT-derived high-fidelity FE simulations with reduced-order models and multi-fidelity Gaussian process (MF-GP) surrogates. The framework is calibrated and validated using experimentally derived porcine ventilator pressure–flow data, enabling rapid and uncertainty-aware estimation of respiratory compliance and resistance while preserving physiological fidelity. By combining high-resolution modeling with computational efficiency, this approach establishes a scalable pathway to translate preclinical insights into patient-specific clinically relevant lung mechanics models. Table 1 contains a summary of the literature on lung mechanics modeling, inverse analysis, and porcine studies, highlighting existing gaps and the contribution of this work.

In summary, this study makes three key contributions. First, we show that multi-fidelity Gaussian process (MF-GP) surrogates enable accurate and uncertainty-aware prediction of respiratory compliance (${ C}_{\textrm{rs}}$) and resistance (${ R}_{\textrm{rs}}$) with errors below 5% relative to high-fidelity simulations, while achieving computational speedups of over five orders of magnitude. Second, we demonstrate that MF-GP outperforms neural networks in data-scarce regimes typical of computational biomechanics. Third, global sensitivity analysis reveals a clear mechanistic separation of parameter influence, with compliance governed by elastic and boundary effects and resistance dominated by permeability, supporting robust parameter identifiability and reduced-order modeling. While similar poroelastic models exist for human lungs, their direct integration with machine learning is not straightforward due to species-specific anatomy, boundary conditions, and mechanical properties. By combining a porcine-specific, CT-derived poroelastic model with a multi-fidelity learning framework, this work provides a computationally efficient and physiologically consistent approach for uncertainty-aware prediction and parameter estimation in lung mechanics.

**Table 1:** Summary of literature on lung mechanics modeling, inverse analysis, and porcine studies, highlighting existing gaps and the contribution of this work

Authors	Topic	Main contributions	Limitations/gaps
Bates and Ht (2009), Kaczka and Dellacá (2011)	Single-compartment respiratory models	Simple resistance–compliance framework for estimating global lung mechanics from ventilator data	Oversimplified; cannot capture nonlinear, heterogeneous, or regional behavior
Badrou et al. (2025)	Poro-hyperelastic FE modeling	Validated FE model with pressure, volume, and strain data	Computationally expensive; not suitable for rapid inference
Manoochehrtayebi et al. (2026)	Microscale poromechanics	Incorporates alveolar mechanics and surface tension effects	Limited scalability to whole-lung or clinical applications
Neelakantan et al. (2022)	Computational lung modeling review	Comprehensive overview of multi-scale and whole-lung approaches	Does not address real-time prediction or surrogate modeling
Berger et al. (2016), Birzle and Wall (2019), Avilés-Rojas and Hurtado (2022), Olabanjo et al. (2025)	Poroelastic FE formulations	Anatomically realistic simulation of lung deformation and fluid–solid coupling	High computational cost limits inverse modeling and UQ
Patte et al. (2022b)	Quasi-static poromechanical model	Captures organ-scale equilibrium deformation	No parameter identification or real-time capability
Peyraut and Genet (2024)	Gravity and heterogeneous pleural pressure modeling	Improved representation of regional ventilation heterogeneity	Focused on forward modeling; lacks efficient inference
Patte et al. (2022a)	Inverse modeling for compliance estimation	Image-based estimation of regional pulmonary compliance	Requires repeated high-fidelity simulations
Laville et al. (2023)	Parameter identifiability analysis	Evaluates sensitivity to optimization formulations	No acceleration for real-time or large-scale use
Peyraut and Genet (2025)	Probabilistic inverse UQ framework	Digital twin approach with uncertainty quantification	Computationally expensive due to reliance on FE simulations
Gerard et al. (2020); Barahona et al. (2024)	Multi-fidelity and ML-based modeling	Integration of physics-based models with machine learning	Primarily focused on human lungs; limited porcine application
Henry and Royston (2018), Peng et al. (2016), Dai et al. (2014b), Dai et al. (2014a)	Porcine poroelastic modeling (high-frequency)	Biot-based modeling of acoustic and vibrational lung mechanics	Restricted to high-frequency, small-amplitude regimes
Yoshida et al. (2018), Giffin et al. (2025), Ruiz (2025)	Porcine models in VILI studies	Establish porcine lungs as key experimental model	Lack integration with advanced computational frameworks
Judge et al. (2014), Suki et al. (2005), Suki and Bates (2025)	Comparative lung physiology	Demonstrate anatomical and functional similarity between porcine and human lungs	Differences in stiffness limit direct model transferability
This Work	Multi-fidelity poroelastic FE–ML framework for porcine lungs	Hybrid HF–LF FE + ML surrogate enabling fast, uncertainty-aware prediction of $C_{rs}$ and $R_{rs}$ (a speed-up of over five orders of magnitude, <5% error)	Relies on global metrics; lacks spatial validation; limited investigation of patient-specific inverse identifiability

## Materials and methods

### Overview of the numerical pipeline

**Fig. 1:**
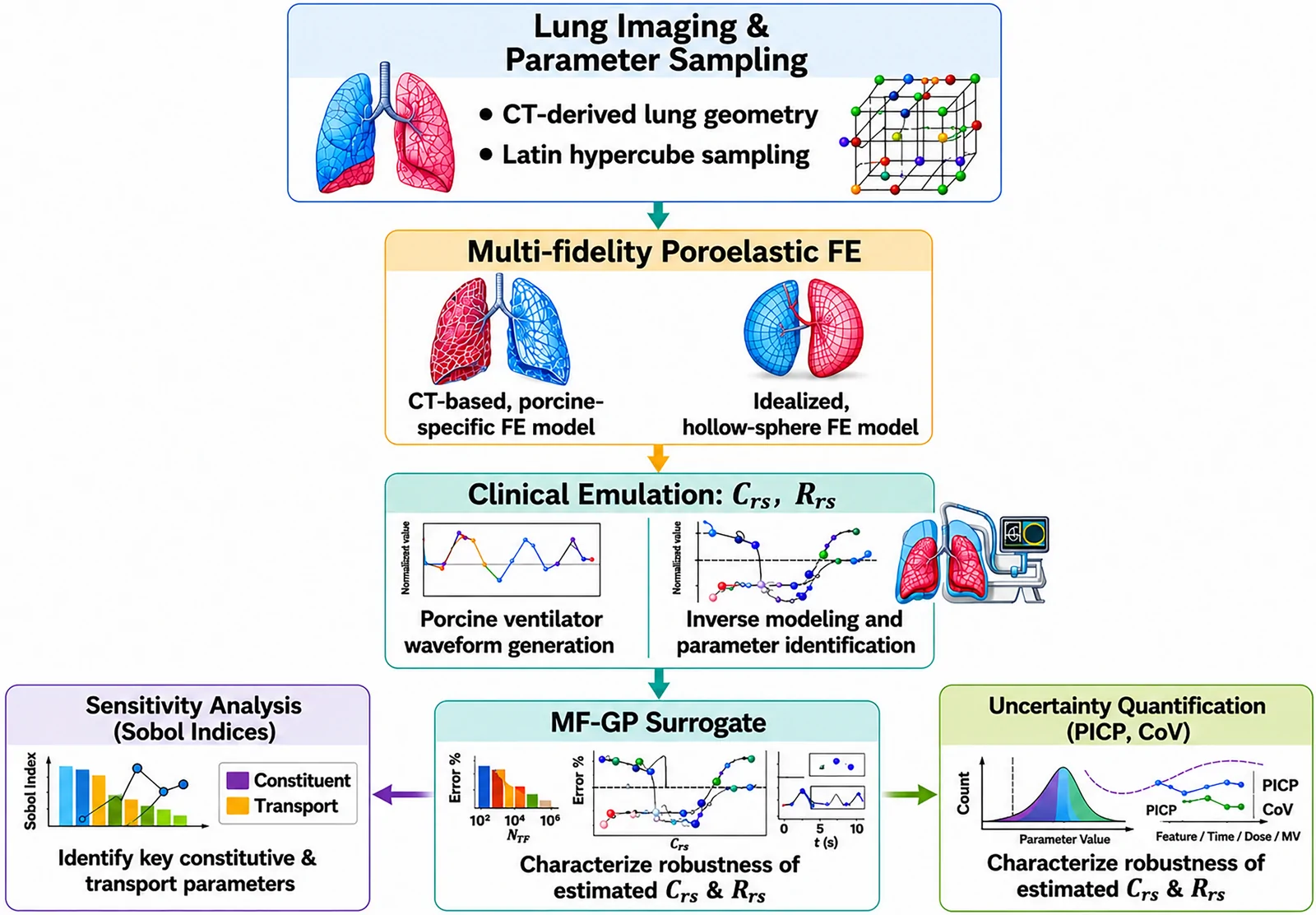
Integrated workflow for poroelastic–ML characterization of lung mechanics. This framework bridges image-derived biomechanics, clinical emulation, and data-driven surrogate modeling to enable sensitivity analysis and uncertainty quantification at minimal computational cost

The overall workflow of this study is illustrated in Fig. 1. Beginning with CT-derived porcine lung imaging, a physiologically relevant parameter space is constructed and sampled to drive a set of poroelastic FE simulations. Two complementary models are employed: a *high-fidelity (HF)* model based on anatomically accurate lung geometry and fully nonlinear poromechanics, and a computationally efficient *low-fidelity (LF)* hollow-sphere model that preserves total lung volume and airway inlet area. Both models are subjected to identical time-dependent physiological boundary conditions, such as prescribed airway pressure, ensuring consistency across fidelity levels. For both HF and LF models, each simulation produces synthetic pressure, volume, and flow waveforms, from which global respiratory mechanics, ${ C}_{\text {rs}}$ and ${ R}_{\text {rs}}$, are extracted using a standard single-compartment inverse model that mirrors clinical pulmonary function assessment. The paired HF and LF outputs are then fused within the MF-GP framework, where LF simulations capture global trends and sparse HF data correct systematic bias. The resulting surrogate enables rapid, differentiable, and uncertainty-aware prediction of ${ C}_{\text {rs}}$ and ${ R}_{\text {rs}}$. Building on the MF-GP emulator, uncertainty quantification is performed to assess the propagation of parametric variability through the surrogate, using prediction interval coverage probability (PICP) and coefficient of variation (CoV) as robustness metrics. In parallel, global sensitivity analysis based on Sobol indices is conducted to identify the poroelastic parameters that most strongly influence compliance and resistance, thereby elucidating the dominant mechanical drivers underlying global lung function.

### Lung geometry acquisition and mesh generation

**Fig. 2:**
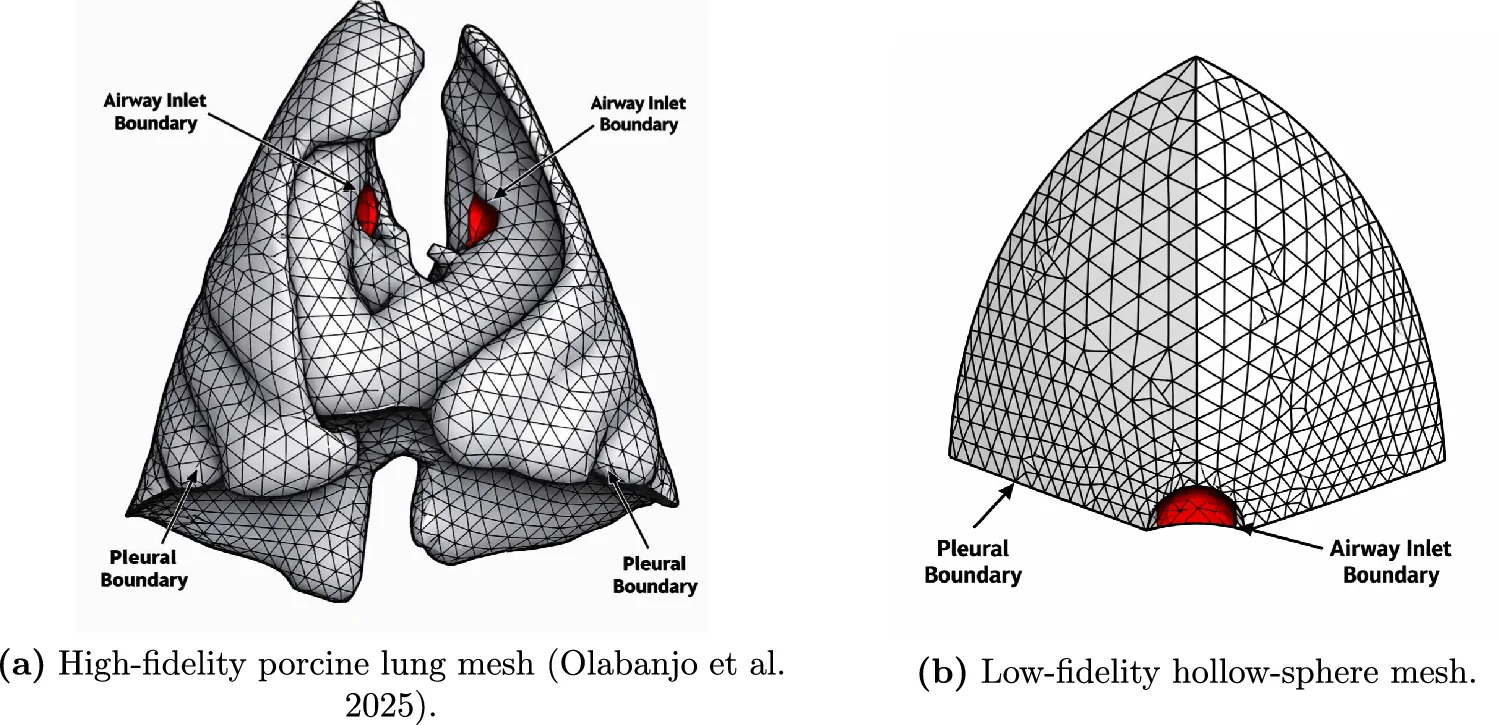
Finite-element meshes for high- and low-fidelity poroelastic simulations

To enable multi-fidelity finite-element (FE) simulations, we constructed two models based on distinct geometric and discretization levels (Fig. 2): a high-fidelity (HF) model and a low-fidelity (LF) model. The HF model is derived from CT-based porcine lung geometry and preserves detailed anatomical structure, enabling accurate resolution of regional deformation and airflow. It is used as the reference model for calibration and validation. In contrast, the LF model employs a simplified, homogenized geometry with a coarser mesh, designed to capture the dominant global pressure–volume (PV) behavior while neglecting fine-scale anatomical detail (Bates and Ht 2009; Suki and Bates 2011). The LF model is introduced to mitigate the high computational cost of HF simulations, which limits their use in large-scale parameter estimation and uncertainty quantification. While HF simulations require hours per run, the LF model can be evaluated in seconds to minutes, enabling efficient exploration of the parameter space. Within the multi-fidelity framework, the LF model provides dense, low-cost predictions, while the HF model supplies sparse, high-accuracy data. These are combined using multi-fidelity Gaussian process (MF-GP) regression to correct LF predictions based on HF information, achieving high accuracy at substantially reduced computational cost (Kennedy and O’Hagan 2000; Perdikaris et al. 2017).

The HF geometry was reconstructed from volumetric computed tomography (CT) scans of an in vivo porcine lung (Siemens SOMATOM Force; 120 kVp, 90 mAs, 0.5 mm slice thickness) acquired at airway pressures ranging from 30 to 0 cmH_2_O. The porcine specimen used in this study was male, with a body weight of 9.1 kg. The exact age of the specimen was not available. The study protocol was approved by the University of Iowa Institutional Animal Care and Use Committee (No. 5061428). Lung parenchyma segmentation was performed using a convolutional neural network adapted from human to porcine anatomy (Gerard et al. 2020). The resulting surface mesh was converted into a volumetric tetrahedral mesh using FEBio. FEBio was used solely for mesh generation, providing high-quality tetrahedral discretization of the complex anatomical geometry. The generated mesh (19,062 elements, 6,066 nodes) was exported in .msh format and subsequently converted into FEniCS-compatible formats (.xdmf, .h5) using Python-based tools within the FEniCS framework. Boundary regions, including the airway inlet and pleural surface, were identified and labeled using ParaView to enable the application of physiologically consistent boundary conditions.

To reduce computational cost while preserving key anatomical features, the LF geometry was constructed as a hollow sphere with total volume and airway inlet area matched to the HF model. Exploiting octant symmetry, a one-eighth spherical section (1,178 elements, 370 nodes; Fig. 2) was generated in Gmsh. The inner radius was set to $r_i = 0.468~\text {cm}$, corresponding to the HF airway cross-sectional radius, and the outer radius $r_o$ was determined to match the total lung volume $V_{\text {lung}} = 3000~\text {cm}^3$. The outer radius was computed as

2 $$\begin{aligned} r_o = \left( \frac{3 V_{\text {lung}}}{4\pi } + r_i^3 \right) ^{1/3}, \end{aligned}$$

yielding $r_o \approx 8.95~\text {cm}$. This construction preserves the overall lung volume while dramatically reducing mesh complexity for computational efficiency. All simulations were performed in the FEniCS environment, where the lung was modeled as a nonlinear poroelastic medium.

### Poroelastic finite-element model and implementation

The lung parenchyma is modeled as a biphasic, nonlinear poroelastic medium following Biot’s theory (Biot 1941) and the poromechanics formulation of  Birzle et al. (2019); Olabanjo et al. (2025); Birzle et al. (2018); Birzle and Wall (2019). The model is defined in the reference (Lagrangian) configuration $\Omega _0 \subset \mathbb {R}^3$ over a physiological time interval t∈(0,T], with primary unknowns: solid displacement $\boldsymbol{u}(\boldsymbol{X},t)$, interstitial fluid pressure $p(\boldsymbol{X},t)$, and porosity $\varphi (\boldsymbol{X},t)$.

#### Model assumptions and governing equations

The poroelastic formulation of the lung parenchyma is based on the following assumptions:

1. **Biphasic medium.** The lung parenchyma is modeled as a biphasic porous medium consisting of a deformable solid skeleton (alveolar tissue) and a single fluid phase representing air. The air is treated as a continuum fluid occupying the pore space of the tissue. Liquid phases (e.g., blood or lining fluid) and multiphase effects are neglected.
2. **Continuum homogenization.** The complex alveolar microstructure is not modeled explicitly. Instead, it is homogenized into an effective continuum, whereby the discrete alveoli and airspaces are represented by averaged macroscopic properties (e.g., effective elasticity, permeability, and porosity). This allows the lung tissue to be treated as a continuous hyperelastic porous material at the organ scale.
3. **Darcy flow in deforming porous media.** Airflow through the deforming porous tissue is governed by Darcy’s law in the reference configuration:
  $$ \boldsymbol{W} = - J \boldsymbol{F}^{-1} \frac{\boldsymbol{K}}{\eta } \boldsymbol{F}^{-T} \nabla _0 p, $$
  where $\boldsymbol{W}$ is the fluid flux, $\boldsymbol{K}$ is the permeability tensor (taken as isotropic, $\boldsymbol{K} = k\boldsymbol{I}$), *k* is the intrinsic permeability, η is the fluid viscosity, and $\nabla _0 p$ is the pressure gradient. We also assume that flow is diffusion-dominated and the viscous forces and inertial effects are negligible.
4. **Quasi-static conditions.** Inertial effects are neglected in both the solid and fluid phases, which is justified by the relatively slow breathing rates compared to elastic wave speeds in lung tissue. The governing equations therefore reduce to a quasi-static equilibrium problem.
5. **Finite-strain kinematics.** Large deformations of the lung parenchyma (typically exceeding 20% strain during normal ventilation) are captured using a nonlinear finite-strain framework. The deformation gradient $\boldsymbol{F}$ and its determinant *J* describe local deformation and volume change.
6. **Single-phase saturation.** The pore space is assumed to be fully occupied by a single fluid phase (air), and multiphase phenomena such as air–liquid interfaces and surface tension effects are neglected. This assumption implies that capillary and interfacial effects are not explicitly modeled.
7. **Isotropic effective response.** The homogenized lung tissue is assumed to exhibit isotropic mechanical behavior at the macroscopic scale. Fluid–solid coupling is governed by standard poroelastic theory (via pressure and deformation), without introducing additional slip or interface conditions. Surfactant effects and microscale anisotropy are neglected.

Under these assumptions, the coupled governing equations in the reference configuration $\Omega _0 \times (0,T]$ are:

**Solid momentum balance:**

3 $$\begin{aligned} -\nabla _0 \cdot \boldsymbol{P} = \boldsymbol{0}, \quad \boldsymbol{P} = \boldsymbol{P}^{\text {eff}} - \alpha p J \boldsymbol{F}^{-T}, \end{aligned}$$

where $\boldsymbol{P}$ is the first Piola–Kirchhoff stress tensor, $\boldsymbol{P}^{\text {eff}}$ is the effective solid stress, and the second term ($\alpha p J \boldsymbol{F}^{-T}$) represents fluid pressure contribution via Biot-type coupling.

The deformation gradient is defined as

$$ \boldsymbol{F} = \boldsymbol{I} + \nabla _0 \boldsymbol{u}, \quad J = \det (\boldsymbol{F}), $$

where $\boldsymbol{u}$ is the displacement field, $\boldsymbol{F}$ is the deformation gradient, $\boldsymbol{I}$ is the identity tensor, and *J* is the Jacobian determinant representing the local volume change. The Biot coefficient $\alpha \in (0,1]$ characterizes the strength of fluid–solid coupling and is taken as α=1 for fully saturated lung tissue.

**Fluid mass conservation:**

4 $$\begin{aligned} \frac{\partial (J \varphi )}{\partial t} - \nabla _0 \cdot \boldsymbol{W} = 0 \end{aligned}$$

where φ is the porosity (fluid volume fraction), and $\boldsymbol{W}$ is the fluid flux in the reference configuration.

**Kinematic porosity relation:**

5 $$\begin{aligned} \varphi = 1 - \frac{1 - \varphi _0}{J}, \end{aligned}$$

where $\varphi _0$ is the initial porosity in the reference configuration and was set to 0.85, consistent with physiologically realistic values reported for lung tissue (Jyoti and Junaid 2020). Equation (5) follows from the assumption of an incompressible solid skeleton, such that all volume changes (captured by *J*) are attributed to variations in pore volume. This corresponds to a quasi-incompressible poroelastic formulation.

Initial conditions are

$$\begin{aligned} \boldsymbol{u}(\boldsymbol{X},0)&= \boldsymbol{0}, \\ p(\boldsymbol{X},0)&= 0. \end{aligned}$$

The boundary of the reference domain, $\partial \Omega _0$, is decomposed into disjoint portions associated with the solid and fluid subproblems. For the solid phase,

$$ \partial \Omega _0 = \Sigma _D \cup \Sigma _N \cup \Sigma _R, \qquad \Sigma _D \cap \Sigma _N = \Sigma _D \cap \Sigma _R = \Sigma _N \cap \Sigma _R = \varnothing , $$

where $\Sigma _D$ denotes the part of the boundary on which displacement is prescribed, $\Sigma _N$ denotes the part on which external surface tractions are applied, and $\Sigma _R$ denotes the part on which a Robin-type elastic support condition is imposed to model lung–ribcage tethering. Let $\boldsymbol{N}$ be the outward unit normal to $\partial \Omega _0$ in the reference configuration. The solid boundary conditions are

$$\begin{aligned} & \boldsymbol{u}= \boldsymbol{u}_D(\boldsymbol{X},t) &  \text {on } \Sigma _D, \\ & \boldsymbol{P}\boldsymbol{N}= \boldsymbol{t}_\Sigma &  \text {on } \Sigma _N, \\ &\boldsymbol{P}\boldsymbol{N}= K_{\textrm{cw}}(\boldsymbol{u}-\boldsymbol{u}_r) &  \text {on } \Sigma _R, \end{aligned}$$

where $\boldsymbol{u}_D$ is the prescribed displacement, $\boldsymbol{t}_\Sigma$ is the prescribed traction per unit reference area, $K_{\textrm{cw}}$ is the Robin stiffness parameter, and $\boldsymbol{u}_r$ is the reference displacement of the surrounding support, taken here as $\boldsymbol{u}_r=\boldsymbol{0}$. For the fluid phase, the boundary is partitioned as

$$ \partial \Omega _0 = \Gamma _D \cup \Gamma _N, \qquad \Gamma _D \cap \Gamma _N = \varnothing , $$

where $\Gamma _D$ is the airway inlet boundary and $\Gamma _N$ is the impermeable pleural surface. The fluid boundary conditions are

$$\begin{aligned} &p= \bar{p}(t) &  \text {on } \Gamma _D, \\ & \boldsymbol{W}\cdot \boldsymbol{N}= 0 &  \text {on } \Gamma _N, \end{aligned}$$

where $\bar{p}(t)$ is the prescribed airway pressure at the inlet.

In the momentum balance equation (3), the hyperelastic constitutive behavior of the lung parenchyma is described using the formulation of Birzle et al. (2019) and Birzle and Wall (2019):

$$ \boldsymbol{P}^{\textrm{eff}} = \frac{\partial E}{\partial \boldsymbol{F}}, \quad E(\boldsymbol{C}) = c(I_1 - 3) + \frac{c}{\beta }(I_3^{-\beta } - 1) + c_1 (I_1 I_3^{-1/3} - 3)^{d_1} + c_3 (I_3^{1/3} - 1)^{d_3}, $$

where $\boldsymbol{C} = \boldsymbol{F}^T \boldsymbol{F}$ is the right Cauchy–Green deformation tensor, $I_1 = \textrm{tr}(\boldsymbol{C})$, $I_3 = \det (\boldsymbol{C})$, and the exponents are fixed as $d_1 = d_3 = 3$. The key parameters of the model include the hyperelastic constants $(c, \beta , c_1, c_3)$, the baseline permeability *k*, and the stiffness of the chest-wall $K_{\textrm{cw}}$.

**Physical Model Parameter Calibration**

Physical model parameters were determined from literature and calibrated against experimental pressure–volume data to ensure physiologically realistic behavior (Akor et al. 2025). The parameters $\beta , c_1, c_3$, and Kcw were adopted from previous lung mechanics studies (Birzle and Wall 2019; Avilés-Rojas and Hurtado 2022; Barahona et al. 2024), while c and k were calibrated within physiologically plausible ranges to reproduce the observed pressure-volume (PV) response and respiratory dynamics. The values reported in Table 2 represent baseline (nominal) physical model parameter values used in the poroelastic lung model. For the purpose of surrogate modeling and uncertainty quantification, a subset of parameters $(c, \beta , c_1, c_3, k, K_{\textrm{cw}})$ is treated as variable and sampled using Latin hypercube sampling (LHS) within the ranges specified in Table 3. The remaining parameters are held fixed at their baseline values.

**Table 2:** Physical model parameter values

Parameter	Value	Description
α	1.0	Biot’s coefficient
$\varphi _0$	0.85	Initial porosity
η	1.83 $\times 10^{-5}$ Pa s	Fluid viscosity
*k*	3.0 $\times 10^{4} \textrm{mm}^{2}$/kPa s	Baseline permeability
$K_{\textrm{cw}}$	0.08 kPa/mm	Chest-wall stiffness
*c*	0.535 kPa	Elastic stiffness
β	1.075	Nonlinearity exponent
$c_1$	0.2782 kPa	Nonlinear elasticity coeff. 1
$c_3$	5.766$\times 10^{-3}$ kPa	Nonlinear elasticity coeff. 3
$d_1, d_3$	3	Viscoelastic exponents

Numerically, the coupled poroelastic system defined in Eqs. (3),  (4) and  (5) is implemented in FEniCS (Alnæs et al. 2011; Barrata et al. 2023; Langtangen et al. 2017) and discretized using Taylor–Hood finite elements, namely piecewise quadratic ($P_2$) elements for the solid displacement $\boldsymbol{u}$ and piecewise linear ($P_1$) elements for the fluid pressure *p*, defined on tetrahedral meshes. As described in Sect. 2.1, two geometric representations are employed: a high-fidelity (HF) CT-derived mesh comprising 19,062 elements, and a low-fidelity (LF) one-eighth hollow-sphere mesh with 1,178 elements. Both geometries are constructed to match the total lung volume and airway inlet radius, ensuring consistency across fidelity levels. Time integration is performed using the backward Euler scheme, and the resulting nonlinear system is solved using Newton–Raphson iterations. At each Newton step, the corresponding linearized system is solved with the MUMPS direct solver, using a relative tolerance of $10^{-6}$ and an absolute tolerance of $10^{-8}$.

For each simulation, the FE solutions are post-processed to compute the simulated lung volume and flow rate as

6 $$\begin{aligned} V_{\text {sim}}(t) = \int _{\Omega _0} J \, \textrm{d}\Omega _0 - V_0, \qquad \dot{V}_{\text {sim}}(t) = \frac{\textrm{d}V_{\text {sim}}}{\textrm{d}t}, \end{aligned}$$

where $V_0$ is the reference lung volume. The global respiratory mechanics-specifically the respiratory compliance ${ C}_{\text {rs}}$ and respiratory resistance ${ R}_{\text {rs}}$—are then estimated by minimizing the discrepancy between the simulated inlet pressure and the pressure predicted by the single-compartment model:

7 $$\begin{aligned} \mathcal {D}({ C}_{\text {rs}}, { R}_{\text {rs}}) = \sum _{i=0}^N \left[ \bar{p}_{\text {sim}}(t_i) - \left( \frac{V_{\text {sim}}(t_i)}{{ C}_{\text {rs}}} +{ R}_{\text {rs}} \dot{V}_{\text {sim}}(t_i) \right) \right] ^2, \end{aligned}$$

where $\bar{p}_{\text {sim}}(t)$ denotes the prescribed airway pressure waveform applied at the inlet.

### Machine learning approaches

The objective of this study is to construct a quantitative mapping between the physical parameters of the poroelastic lung model and the resulting global respiratory mechanics, namely the respiratory compliance ${ C}_{\text {rs}}$ and respiratory resistance ${ R}_{\text {rs}}$. Following the poroelastic FE simulations, these global metrics are obtained from the simulated pressure–volume–flow responses using the inverse procedures defined in Eqs. (6) and (7). While direct use of high-fidelity FE simulations can provide accurate estimates of ${ C}_{\text {rs}}$ and ${ R}_{\text {rs}}$, such an approach is computationally prohibitive for large-scale parametric analysis.

To address this limitation, we develop a machine learning (ML) surrogate model trained on a multi-fidelity dataset generated from poroelastic FE simulations. This surrogate emulates the mapping from poroelastic constitutive parameters to global respiratory mechanics in a manner consistent with the single-compartment model in (1). Specifically, the dataset is defined as

$$ S = \left\{ \big ( {\textbf {q}}^{(i)}, {\textbf {r}}^{(i)} \big ) \right\} _{i=1}^{N}, $$

where each input vector ${\textbf {q}}^{(i)} \in \mathbb {R}^6$ consists of the physical model parameters $(c, \beta , c_1, c_3, k_0, K_{\textrm{cw}})$, and the corresponding output vector ${\textbf {r}}^{(i)} \in \mathbb {R}^2$ contains the reduced/global model parameters: respiratory system compliance ${ C}_{\text {rs}}$ and resistance ${ R}_{\text {rs}}$, obtained via inverse modeling of FE simulations.

To enable multi-fidelity learning, each set of sampled parameters is evaluated using both high-fidelity (HF) and low-fidelity (LF) FE models, producing paired datasets $S^{(\textrm{HF})}$ and $S^{(\textrm{LF})}$. Due to the high computational cost of HF simulations (Smith 2024), only 20 HF training samples were generated. To ensure adequate coverage of the six-dimensional parameter space, 200 LF samples were generated using Latin hypercube sampling (LHS), which provides a space-filling design. Within the multi-fidelity framework, the LF data capture global trends across the parameter space, while the HF data provide local corrections to improve predictive accuracy. An additional 100 samples were reserved for testing and used exclusively for independent validation of the surrogate models. All input parameters were standardized to zero mean and unit variance to improve training stability (Barahona et al. 2024).

Two machine learning models are considered: a feedforward neural network (NN) and a multi-fidelity Gaussian process (MF-GP). Model performance is assessed based on predictive accuracy and uncertainty calibration. The NN and MF-GP models are compared to balance predictive accuracy, computational efficiency, and uncertainty quantification, with the best-performing surrogate selected for subsequent analysis. The best-performing surrogate is subsequently employed for efficient global sensitivity analysis and forward uncertainty propagation. By replacing expensive FE simulations with a fast, differentiable surrogate, this framework enables systematic exploration of how poroelastic parameters influence global lung mechanics at a fraction of the original computational cost.

#### Neural network approach

A feedforward neural network (NN) is employed as a baseline surrogate to approximate the nonlinear mapping from physical (poroelastic) model parameters

$$ {\textbf {q}} = (c, \beta , c_1, c_3, k, K_{\textrm{cw}}) \in \mathbb {R}^6 $$

to the corresponding reduced/global model parameters (global respiratory mechanics),

$$ {\textbf {r}} = ({ C}_{\text {rs}}, { R}_{\text {rs}}) \in \mathbb {R}^2. $$

The NN represents a parametric function $\textrm{NN}({\textbf {q}}; \boldsymbol{\theta }) \approx {\textbf {r}}$, where $\boldsymbol{\theta }$ denotes the collection of trainable weights and biases. All input parameters are standardized prior to training, as described in Sect. 2.4.

The network architecture is selected via grid search over the number of hidden layers (1–4), neurons per layer (32–256), learning rates ($10^{-4}$–$10^{-2}$), and total training epochs (500–5000). Rectified linear unit (ReLU) activation functions are used in the hidden layers to balance expressivity and optimization stability, while a linear activation is applied in the output layer to preserve the continuous scale of the predicted quantities. Neural network (NN) model parameters θ are optimized by minimizing the mean squared error (MSE) loss using the Adam optimizer (Bock and Weiß 2019):

$$ \mathcal {L}(\boldsymbol{\theta }) = \frac{1}{N_t} \sum _{m=1}^{N_t} \left\| \textrm{NN}({\textbf {q}}_m;\, \boldsymbol{\theta }) - \hat{{\textbf {r}}}_m \right\| ^2, $$

where $N_t$ denotes the number of training samples, ${\textbf {q}}_m$ is the vector of input parameters *m*-th, and $\hat{{\textbf {r}}}_m$ is the corresponding target output obtained from the inverse modeling of the poroelastic FE simulations.

To avoid overfitting due to the limited size of the high-fidelity (HF) dataset, several strategies were adopted. First, the neural network architecture was designed with moderate complexity, using a limited number of hidden layers and neurons to balance expressiveness and generalization. Second, regularization techniques, including L2 weight decay and early stopping based on validation loss, were employed during training. These methods prevent the model from fitting noise in the training data. Third, the multi-fidelity framework itself reduces the reliance on HF data by leveraging a larger set of low-fidelity (LF) simulations to capture global trends, while the HF data are used to refine the surrogate predictions. Finally, model performance was evaluated using a validation set and independent test samples. The consistency between training and validation errors, along with low prediction errors on unseen data, indicates that overfitting was effectively controlled.

The predictive performance of the trained NN surrogate is evaluated on an independent test set using the mean squared error (MSE), coefficient of determination ($R^2$), and residual analysis. While the NN provides efficient point predictions, its limited capability for uncertainty quantification motivates the adoption of the multi-fidelity Gaussian process framework described in the following subsection.

#### Multi-fidelity Gaussian process approach

In addition to the neural network surrogate, a multi-fidelity Gaussian process (MF-GP) model is constructed to emulate the mapping from physical (poroelastic) model parameter space ${\textbf {q}} \in \mathbb {R}^6$ to reduced/global model parameters - global lung mechanics outputs ${\textbf {r}} = ({ C}_{\text {rs}}, { R}_{\text {rs}})$, while explicitly exploiting the hierarchical structure of the high- and low-fidelity finite-element simulations introduced earlier. Compared with purely data-driven surrogates, the MF-GP provides a probabilistic framework with built-in uncertainty quantification, which is particularly advantageous in regimes where high-fidelity data are limited  (Barahona et al. 2024; Perdikaris et al. 2017; Willard et al. 2022).

The MF-GP follows the autoregressive formulation of Kennedy and O’Hagan (Kennedy and O’Hagan 2000), in which the high-fidelity response is expressed as a scaled low-fidelity prediction plus a correction term:

$$ f^{HF}({\textbf {q}}) = \rho \, f^{LF}({\textbf {q}}) + \delta ({\textbf {q}}). $$

Here, $\rho \in \mathbb {R}$ is a scalar coefficient that captures the global correlation between fidelity levels, while the discrepancy function $\delta ({\textbf {q}})$ accounts for systematic modeling errors not captured in the low-fidelity simulations. Both $f^{LF}({\textbf {q}})$ and $\delta ({\textbf {q}})$ are modeled as independent Gaussian processes with zero mean:

$$ f^{LF}({\textbf {q}}) \sim \mathcal{G}\mathcal{P}\!\left( 0, k_{LF}({\textbf {q}}, {\textbf {q}}')\right) , \quad \delta ({\textbf {q}}) \sim \mathcal{G}\mathcal{P}\!\left( 0, k_{\delta }({\textbf {q}}, {\textbf {q}}')\right) . $$

Squared-exponential covariance kernels are adopted for both components,

$$ k_{\ell }({\textbf {q}}, {\textbf {q}}') = \sigma _{\ell }^2 \exp \!\left( -\sum _{j=1}^{6} \frac{(q_j - q_j')^2}{2 \ell _{\ell ,j}^2} \right) , \quad \ell \in \{\textrm{LF}, \delta \}, $$

where $\sigma _{\ell }^2$ and $\ell _{\ell ,j}$ denote the signal variance and characteristic length scales, respectively. Together with the autoregressive coefficient ρ, these quantities form the set of hyperparameters learned from data.

Given a combined training dataset consisting of low- and high-fidelity simulation outputs, the MF-GP induces a joint Gaussian prior whose structured covariance encodes both within-fidelity smoothness and cross-fidelity correlation. Hyperparameters are estimated by maximizing the marginal log-likelihood. For any unseen parameter realization ${\textbf {q}}^*$, the posterior predictive distribution remains Gaussian,

$$ p({\textbf {r}}^*\mid {\textbf {q}}^*, S) = \mathcal {N}(\boldsymbol{\mu }^*, \boldsymbol{\Sigma }^*), $$

yielding not only point predictions but also calibrated uncertainty estimates.

### Physical model parameter selection and ranges

The parameter ranges summarized in Table 3 were selected based on literature values and physiologically plausible variability in porcine lung tissue. The constitutive parameters $(c, \beta , c_1, c_3)$ are consistent with previously reported nonlinear elastic models of lung parenchyma (Birzle et al. 2018; Barahona et al. 2024). The baseline values correspond to nominal parameter estimates, while the ranges were defined to account for inter-subject variability and uncertainty, typically within $\pm 50\%$ of the baseline values. Similarly, the chest-wall effect $K_{\textrm{cw}}$ and permeability parameter *k* were selected based on reported ranges in respiratory mechanics and poroelastic modeling studies (Barahona et al. 2024). These bounds ensure that the parameter space remains physiologically realistic while enabling robust uncertainty quantification.

**Table 3:** Baseline and range values of physical model parameters used in the lung mechanics simulations

Parameter	Range	Baseline value	Unit
*c*	0.2675–0.8025	0.535	kPa
β	0.5375–1.6125	1.075	–
$c_1$	0.1391–0.4173	0.2782	kPa
$c_3$	2.883 $\times 10^{-3}$–8.649 $\times 10^{-3}$	$5.766 \times 10^{-3}$	kPa
$K_{\textrm{cw}}$	0.04–0.12	0.08	kPa mm$^{-1}$
*k*	1.5 $\times 10^{4}$–4.5 $\times 10^{4}$	$3.0 \times 10{4}$	mm$^{2}$ kPa$^{-1}$ s

### Sensitivity and uncertainty analysis

To interpret the MF-GP surrogate and quantify the influence of poroelastic parameters on global lung mechanics, we conduct a global sensitivity analysis based on Sobol indices (Sobol 2001). The surrogate defines a mapping from the six-dimensional parameter space ${\textbf {q}} \in \mathbb {R}^6$ to the outputs ${\textbf {r}} = ({ C}_{\text {rs}}, { R}_{\text {rs}})^\top$. For each output, the first-order Sobol index $S_i$ measures the direct contribution of parameter $x_i$ to output variance, while the total-effect index $S_{T_i}$ captures both main and interaction effects:

$$ S_i = \frac{\textrm{Var}\!\left( \mathbb {E}[{\textbf {r}}\mid q_i]\right) }{\textrm{Var}({\textbf {r}})}, \qquad S_{T_i} = 1 - \frac{\textrm{Var}\!\left( {\textbf {r}}\mid q_{\sim i}\right) }{\textrm{Var}({\textbf {r}})}, $$

where $q_{\sim i}$ denotes all parameters except $q_i$. The difference $S_{T_i}-S_i$ therefore quantifies higher-order interactions. Sobol indices are estimated using the Saltelli sampling scheme implemented in SALib (Herman and Usher 2017; Saltelli 2002), with $N=10{,}000$ base samples, resulting in 140, 000 surrogate evaluations. All model queries are performed using the MF-GP, thereby avoiding additional finite-element simulations. To further visualize parameter effects, the main-effect function $M^{\textrm{eff}}(q_i)=\mathbb {E}[{\textbf {r}}\mid q_i]-\mathbb {E}[{\textbf {r}}]$ is computed by averaging over 100 Latin hypercube samples spanning the parameter ranges in Table 3 while varying $q_i$.

Uncertainty quantification is performed in two complementary settings: forward uncertainty propagation and predictive uncertainty assessment. For forward UQ, input parameters are assumed to follow independent uniform distributions over the prescribed ranges. A Latin hypercube ensemble of $N=10{,}000$ samples $\{\boldsymbol{x}^{(i)}\}$ is propagated through the MF-GP, yielding predictive distributions

$$ p(r \mid {\textbf {q}}^{(i)}) = \mathcal {N}\!\left( \mu ^{(i)}, \sigma ^{2(i)}\right) . $$

Sampling from these distributions produces an ensemble approximation of the marginal output distribution, capturing both parametric uncertainty and surrogate-induced epistemic uncertainty in a fully non-intrusive manner.

Predictive UQ is assessed on an independent high-fidelity test set. For each test input ${\textbf {q}}_*$, we evaluate the posterior mean $\mu ({\textbf {q}}_*)$, posterior variance $\sigma ^2({\textbf {q}}_*)$, and the corresponding 95% prediction interval. Model calibration is quantified using the prediction interval coverage probability (PICP), defined as the fraction of high-fidelity reference solutions lying within the predicted intervals. Consistent coverage near the nominal level, together with low predictive bias, indicates a well-calibrated and reliable surrogate (Smith 2024; Gardner et al. 2014).

### Evaluation metrics for the ML approaches

Surrogate accuracy, agreement, and uncertainty calibration are evaluated using complementary statistical metrics.

Let $\{(r_i,\hat{r}_i)\}_{i=1}^{N}$ denote pairs of high-fidelity (HF) poroelastic finite-element (FE) reference outputs and surrogate predictions for either respiratory compliance (${ C}_{\text {rs}}$) or respiratory resistance (${ R}_{\text {rs}}$). Global predictive accuracy is quantified using the coefficient of determination,

$$ R^2 = 1 - \frac{\sum _{i=1}^{N} (r_i - \hat{r}_i)^2}{\sum _{i=1}^{N} (r_i - \bar{r})^2}, $$

where $\bar{r}=\frac{1}{N}\sum _{i=1}^{N}r_i$. The $R^2$ metric measures the fraction of HF variance explained by the surrogate, with values near unity indicating strong agreement. Absolute error is assessed via the root mean square error (RMSE),

$$ \textrm{RMSE}=\left( \frac{1}{N}\sum _{i=1}^{N}(r_i-\hat{r}_i)^2\right) ^{1/2}, $$

which penalizes large deviations and is expressed in the physical units of each output.

Agreement beyond correlation-based measures is examined using Bland–Altman analysis. Defining the difference and mean as

$$ d_i=\hat{r}_i-r_i, \qquad m_i=\frac{\hat{r}_i+r_i}{2}, $$

the mean bias $\mu _d$ and 95% limits of agreement,

$$ \mu _d \pm 1.96\,\sigma _d, $$

with $\sigma _d$ the standard deviation of $\{d_i\}$, characterize systematic bias and dispersion across the physiological range.

For probabilistic surrogates, uncertainty calibration is evaluated using the prediction interval coverage probability (PICP). Given posterior predictions $p(r_i\mid {\textbf {q}}_i)=\mathcal {N}(\mu _i,\sigma _i^2),$ the PICP at confidence level (1-α) is

$$ \textrm{PICP}=\frac{1}{N}\sum _{i=1}^{N} \mathbb {I}\!\left( |r_i-\mu _i|\le z_{\alpha /2}\sigma _i\right) , $$

where $z_{\alpha /2}$ is the standard normal quantile. A well-calibrated surrogate satisfies $\textrm{PICP}\approx 1-\alpha$.

Relative predictive uncertainty is summarized by the coefficient of variation (CoV),

$$ \textrm{CoV}=\frac{\sigma _r}{\mu _r}, $$

where $\mu _r$ and $\sigma _r$ are the mean and standard deviation of the predicted output distribution.

Finally, the numerical reliability of global sensitivity analysis is verified by monitoring the convergence of Sobol indices. Convergence is achieved when successive estimates satisfy

$$ |S_i^{(N)}-S_i^{(N+\Delta N)}|<\varepsilon , $$

for a prescribed tolerance ε, ensuring that reported sensitivities are not contaminated by sampling error.

Together, these metrics provide a consistent and uncertainty-aware framework for assessing surrogate fidelity, agreement with physics-based simulations, and robustness of sensitivity inference.

## Results

This section presents results from poroelastic finite-element simulations based on Biot’s theory, a comparative assessment of machine learning approaches, and subsequent global sensitivity analysis and uncertainty quantification.

### Poroelastic finite-element simulations

The poroelastic finite-element simulations were performed to replicate the biomechanical response of lung parenchyma under dynamic loading conditions. To assess model fidelity, we compare simulated pressure, flow, and volume waveforms against experimental measurements and validate the inferred poroelastic parameters against established physiological ranges reported in the literature. The results presented below demonstrate both the quantitative accuracy of the numerical model and its consistency with known lung biomechanics.

#### Experimental versus simulated waveforms

**Fig. 3:**
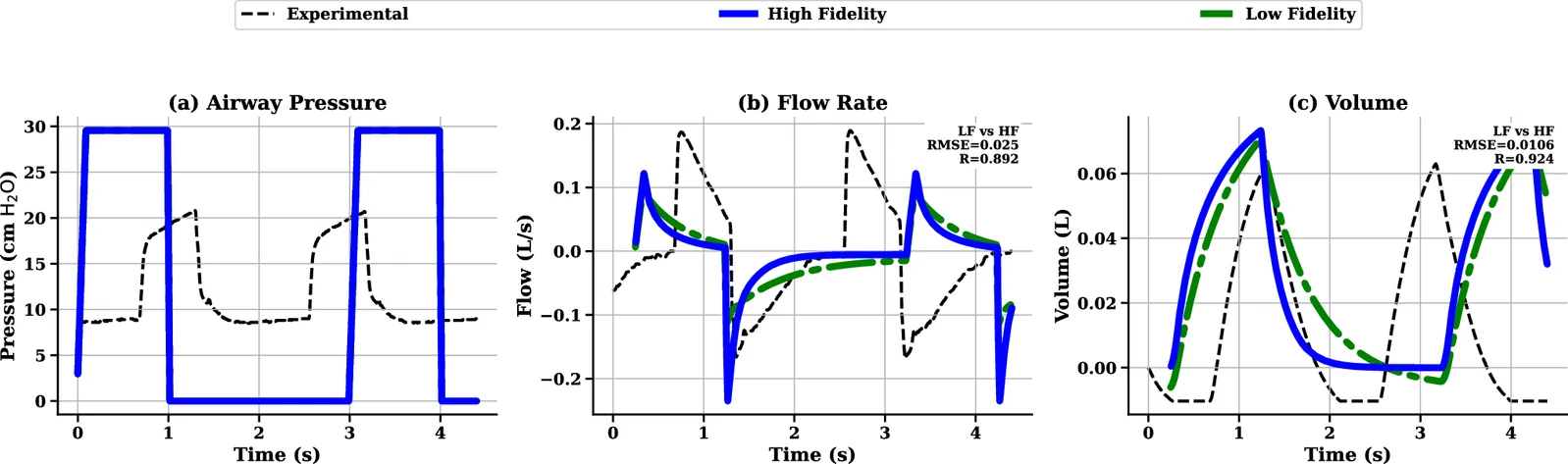
Overlay of experimental and simulated respiratory waveforms: **a** airway pressure, **b** flow rate, and **c** lung volume. The simulated pressure corresponds to the prescribed pressure-controlled ventilation input (0 cmH2O to 30 cmH2O), while flow and volume signals were temporally aligned for comparison

We evaluate the physiological fidelity of the proposed poroelastic finite-element (FE) framework by comparing simulated respiratory waveforms against experimental measurements acquired during pressure-controlled mechanical ventilation. The experimentally measured airway pressure, flow, and volume signals provide clinically relevant benchmarks for validating the simulated respiratory response. Figure 3 presents the overlay of experimental and simulated respiratory waveforms obtained from the high-fidelity (HF) and low-fidelity (LF) poroelastic FE models. In panel (a), the HF and LF simulations overlap almost exactly because both models are driven by the same prescribed pressure-controlled ventilation waveform (0 cmH2O to 30 cmH2O). In contrast, the experimental pressure waveform exhibits smoother transitions and lower peak amplitudes, reflecting physiological damping and ventilator-system dynamics not explicitly represented in the idealized pressure boundary condition.

For the flow waveform (panel (b)), both numerical models reproduce the overall inspiratory–expiratory structure observed experimentally, including inspiratory acceleration and expiratory reversal. The LF model demonstrates strong agreement with the HF solution after temporal alignment and scaling (R=0.892, RMSE =0.025), indicating that the LF formulation preserves the dominant global resistive behavior of the HF model. However, the HF model captures sharper transient peaks and expiratory dynamics, whereas the LF waveform appears smoother and more dissipative, reflecting its reduced anatomical and constitutive resolution. Relative to the experimental measurements, both models reproduce the principal ventilatory dynamics but underestimate certain transient asymmetries and waveform smoothness. The volume waveform (panel (c)) shows stronger agreement between HF and LF than observed for flow. The LF model closely reproduces the HF tidal-volume evolution, yielding high correlation (R=0.924, RMSE =0.0106). Both models capture similar inflation amplitudes, inspiratory timing, and expiratory decay, indicating that the LF model preserves the dominant global compliance characteristics of the HF poroelastic formulation. Compared with the experimental waveform, the HF model more accurately reproduces inspiratory filling and expiratory emptying, while the LF model provides a smoother approximation of the global volume response (Table 4).

**Table 4:** Quantitative comparison between HF and LF simulated respiratory waveforms

Variable	RMSE	Correlation
Flow	0.025	0.892
Volume	0.0106	0.924

Overall, the overlays demonstrate that the HF model more effectively resolves transient respiratory mechanics and localized flow dynamics, whereas the LF model accurately preserves the dominant global compliance and resistance behavior at substantially lower computational cost. Agreement between HF and LF is consistently stronger for volume than for flow, reflecting the integrated nature of volume signals and the greater sensitivity of flow to localized resistive and transient effects. These findings support the LF poroelastic model as an efficient surrogate for estimating global respiratory mechanics, while retaining the HF formulation as the reference standard for resolving finer anatomical and transient mechanical behavior. These results demonstrate that LF simulations provide computationally efficient approximations of global respiratory mechanics while preserving the dominant compliance and resistance behavior of the HF poroelastic model. In contrast, the HF formulation remains necessary for accurately resolving localized transient flow dynamics and anatomically detailed mechanical responses essential for reliable surrogate training and parameter identification of ${ C}_{\textrm{rs}}$ and ${ R}_{\textrm{rs}}$.

#### Validation of predicted lung global parameters against literature values

We compare the predicted respiratory compliance $({ C}_{\text {rs}})$ and respiratory resistance $({ R}_{\text {rs}})$ from our poroelastic finite-element (FE) models with experimentally reported values for porcine subjects in the literature (Herrmann et al. 2020) to evaluate their physiological plausibility. As summarized in Table 5, published studies report static compliance in Juvenile pigs (9–13 kg) to range from 5.68 to 8.20 mL/cm (H2O), and respiratory resistance to fall between 5.7 and 12.1 cm (H2O)s/L.

**Table 5:** Literature ranges of porcine lung mechanical parameters for juvenile subjects (9–13 kg) (Herrmann et al. 2020)

Parameter	Juvenile (9–13 kg)
Respiratory compliance (mL/cmH2O)	5.68 – 8.20
Respiratory resistance (cmH2Os/L)	5.7 – 12.1

**Table 6:** Predicted lung mechanical parameters from poroelastic FE simulations (baseline parameter set)

Parameter	High-fidelity	Low-fidelity
Baseline constitutive parameters	$$\begin{aligned} c&= {0.535}\,{\textrm{kPa}},\; \beta = 1.075,\; c_1 = {0.2782}\,{\textrm{kPa}} \\ c_3&= 5.766 \times 10^{-3}\,{\textrm{kPa}},\; k = 3.0 \times 10^{4} \textrm{mm}^{2}/kPa\, \cdot \, s,\; K_{\textrm{cw}} = 0.08 \textrm{kPa}/\textrm{mm} \end{aligned}$$	
Respiratory compliance (${ C}_{\text {rs}}$)	6.35 mL/cmH2O	6.12 mL/cmH2O
Respiratory resistance (${ R}_{\text {rs}}$)	8.78 cmH2Os/L	8.13 cmH2Os/L
Computation time	1 hr 0 min 42 s (3642.90 s)	2.95 min (177 s)

Using a baseline set of constitutive parameters ($c = {0.535}\,{kPa}$, β=1.075, $c_1 = {0.2782}\,{\textrm{kPa}}$, $c_3 = 5.766 \times 10^{-3} \textrm{kPa}$, $k = 3.0 \times 10^{4} \textrm{mm}^{2}/\textrm{kPa} \cdot \textrm{s}$, $K_{\textrm{cw}} = 0.08 \textrm{kPa}/\textrm{mm}$), our high-fidelity FE simulation predicts a respiratory compliance of ${ C}_{\text {rs}} = 6.35\,\textrm{mL}/\textrm{cmH2O}$ and resistance of ${ R}_{\text {rs}} = 8.78 \textrm{cmH2Os}/\textrm{L}$ (Table  6). The low-fidelity model yields comparable values: ${ C}_{\text {rs}} = 6.12\textrm{mL}/\textrm{cmH2O}$ and ${ R}_{\text {rs}} = 8.13\textrm{cm} \mathrm{(H2O)s}/\textrm{L}$. Both predicted values fall well within the established literature ranges for juvenile pigs, validating the physiological realism of our model.

### Comparison of ML-based approaches

We evaluated the predictive performance of two surrogate modeling approaches—multi-fidelity Gaussian process (MF-GP) and neural network (NN)—by comparing their estimates of respiratory compliance (${ C}_{\text {rs}}$) and respiratory resistance (${ R}_{\text {rs}}$) against high-fidelity (HF) poroelastic finite-element simulations derived from CT-based porcine lung geometries. The selection of MF-GP and NN as surrogate models in this study is motivated by their representation of two different paradigms in scientific machine learning. Multi-fidelity Gaussian processes are nonparametric, probabilistic models that are particularly well-suited for small-data regimes and enable the principled integration of low- and high-fidelity information with inherent uncertainty quantification. In contrast, neural networks are highly flexible, data-driven function approximators that typically require larger datasets to achieve robust generalization, especially in nonlinear settings.

**Fig. 4:**
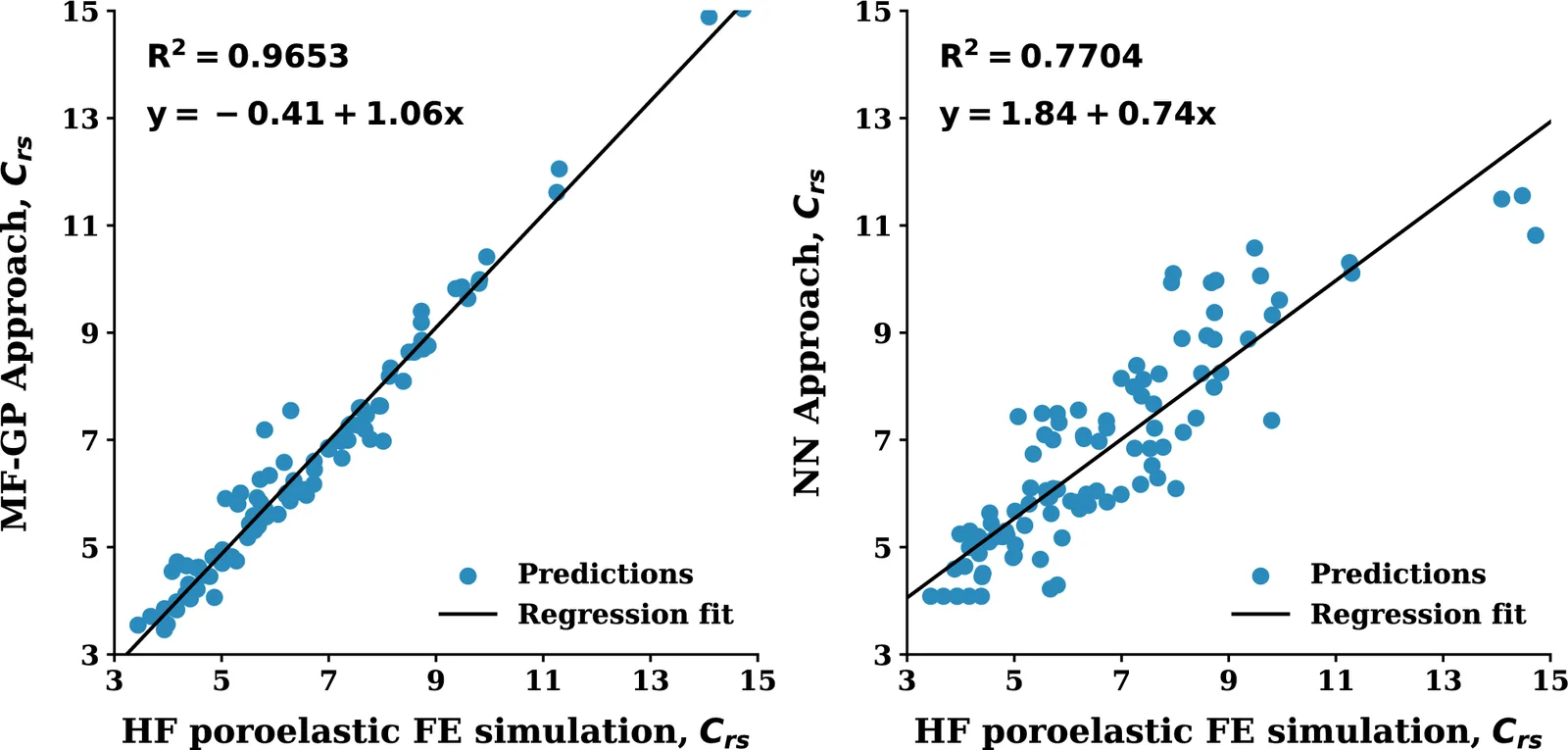
Correlation plots for ${ C}_{\textrm{rs}}$: MF-GP approach (left) and NN approach (right) compared against HF poroelastic FE simulations. The solid line represents the linear regression fit; regression equations and coefficients of determination ($R^2$) are displayed

The comparison between these approaches is therefore not intended solely to identify the better-performing model, but to provide insight into how different surrogate modeling strategies perform under the practical constraint of limited high-fidelity data. This is especially relevant in the context of poroelastic lung simulations, where high-fidelity data are computationally expensive to generate. As such, this analysis highlights important trade-offs between data efficiency, predictive accuracy, and robustness, informing the selection of surrogate models for computational biomechanics applications. It should be noted that the high-fidelity (HF) dataset used in this study is relatively limited (20 samples), reflecting the high computational cost of poroelastic finite-element simulations. Consequently, the comparative performance of surrogate models is evaluated within a data-scarce regime, which may influence the generalizability of the observed trends.

**Fig. 5:**
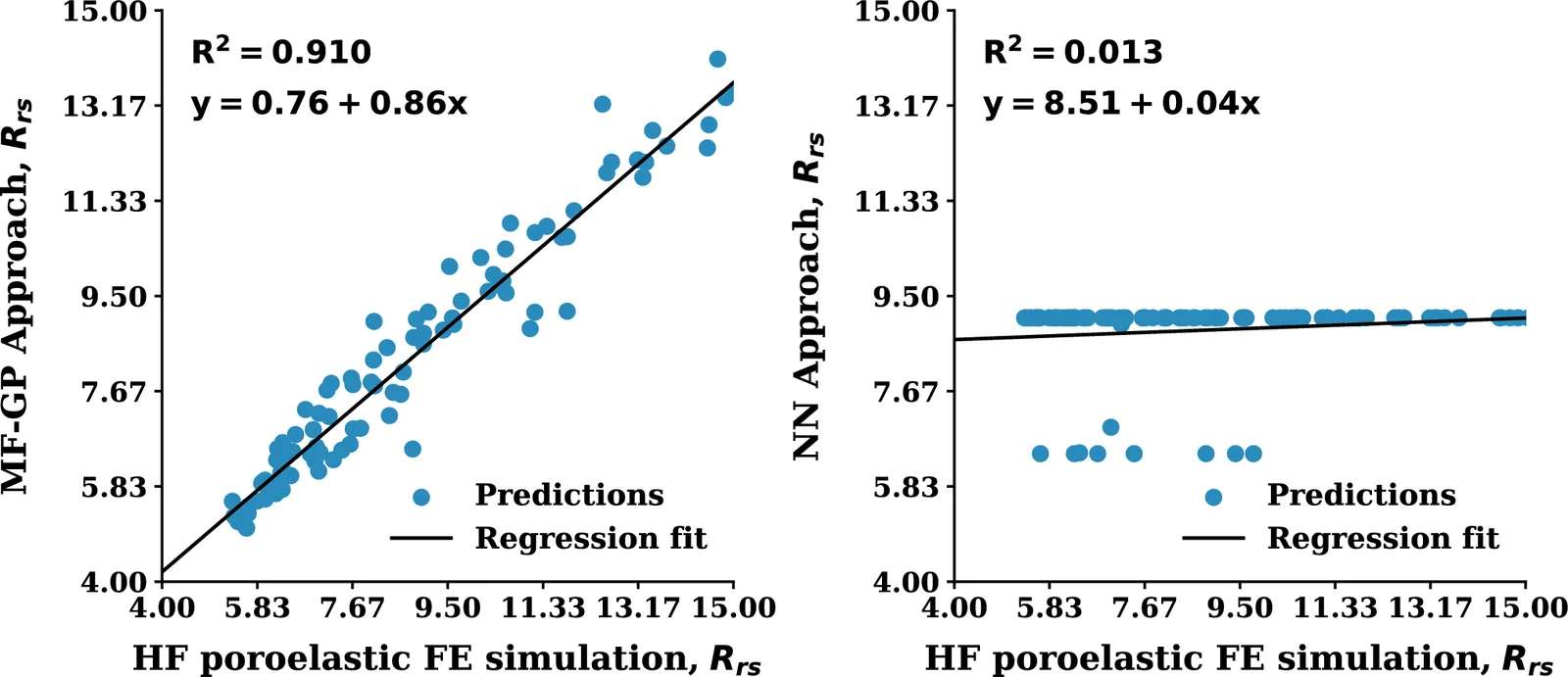
Correlation plots for ${ R}_{\textrm{rs}}$: MF-GP approach (left) and NN approach (right) compared against HF poroelastic FE simulations

Figure 4 demonstrates the predictive performance of the surrogate models for respiratory compliance ${ C}_{\textrm{rs}}$. The MF-GP approach exhibits strong agreement with the HF poroelastic FE simulations, achieving $R^2 = 0.9653$ with regression relation

y=-0.41+1.06x,

indicating excellent preservation of the underlying compliance trend across the physiological range. The regression slope close to unity and small intercept demonstrate minimal systematic bias and near one-to-one agreement with HF predictions. In contrast, the NN approach shows substantially reduced predictive fidelity for ${ C}_{\textrm{rs}}$, with $R^2 = 0.7704$ and regression equation

y=1.84+0.74x.

The reduced slope and increased scatter indicate systematic underestimation at higher compliance values and weaker generalization performance relative to the MF-GP model. For respiratory resistance ${ R}_{\textrm{rs}}$ (Fig. 5), the MF-GP again demonstrates strong predictive capability, achieving $R^2 = 0.910$ with regression equation

y=0.76+0.86x,

indicating close agreement with HF simulations over the investigated physiological range. In contrast, the NN approach fails to capture meaningful resistance variability, yielding an almost constant prediction with very poor agreement ($R^2 = 0.013$),

y=8.51+0.04x.

The near-zero regression slope confirms that the NN model is unable to resolve the dependence of ${ R}_{\textrm{rs}}$ on the underlying poroelastic parameters within the sparse-data regime considered here.

**Fig. 6:**
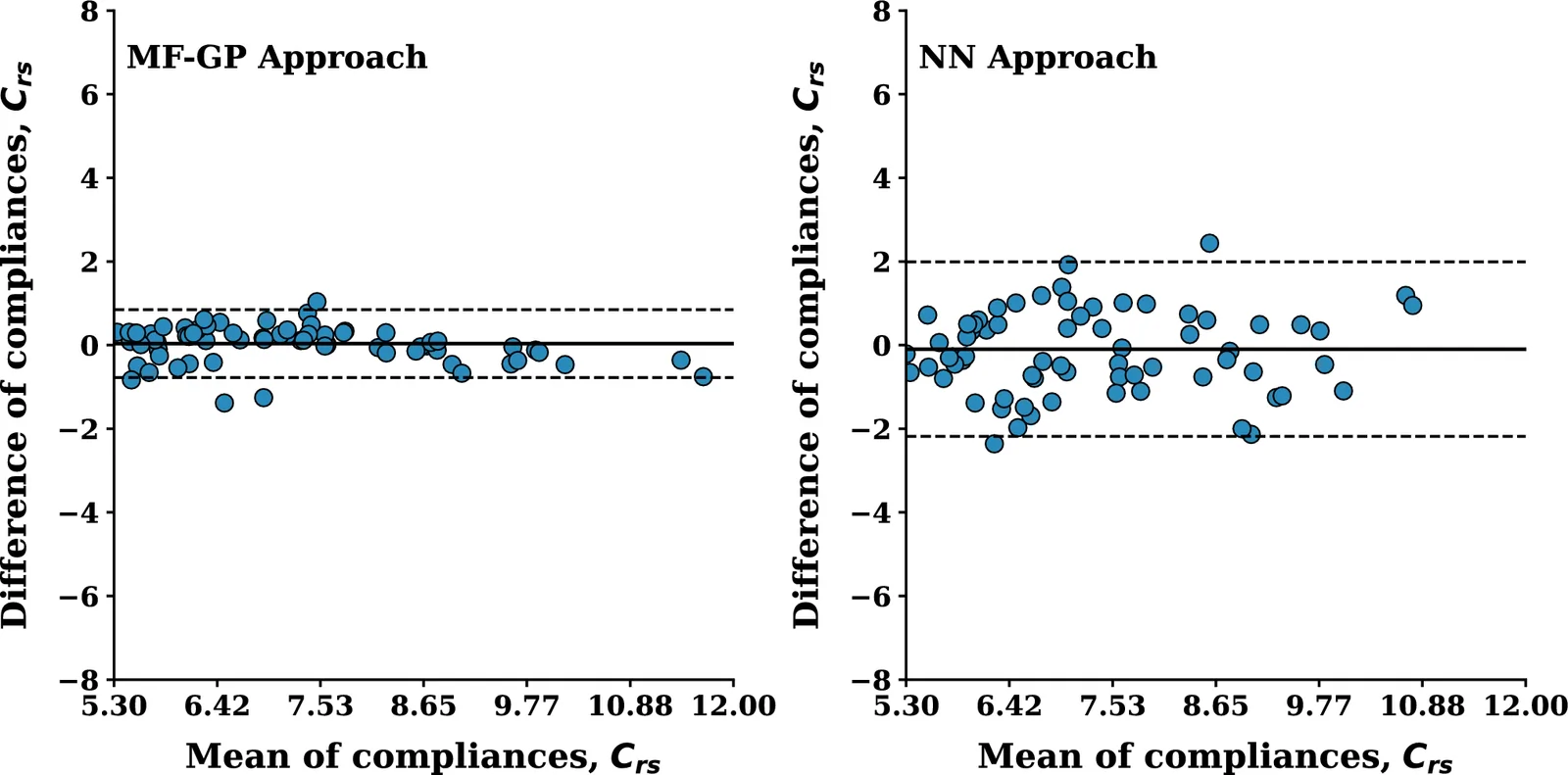
Bland–Altman plots for ${ C}_{\textrm{rs}}$: MF-GP approach (left) and NN approach (right). Solid line = mean bias; dashed lines = 95% limits of agreement

**Fig. 7:**
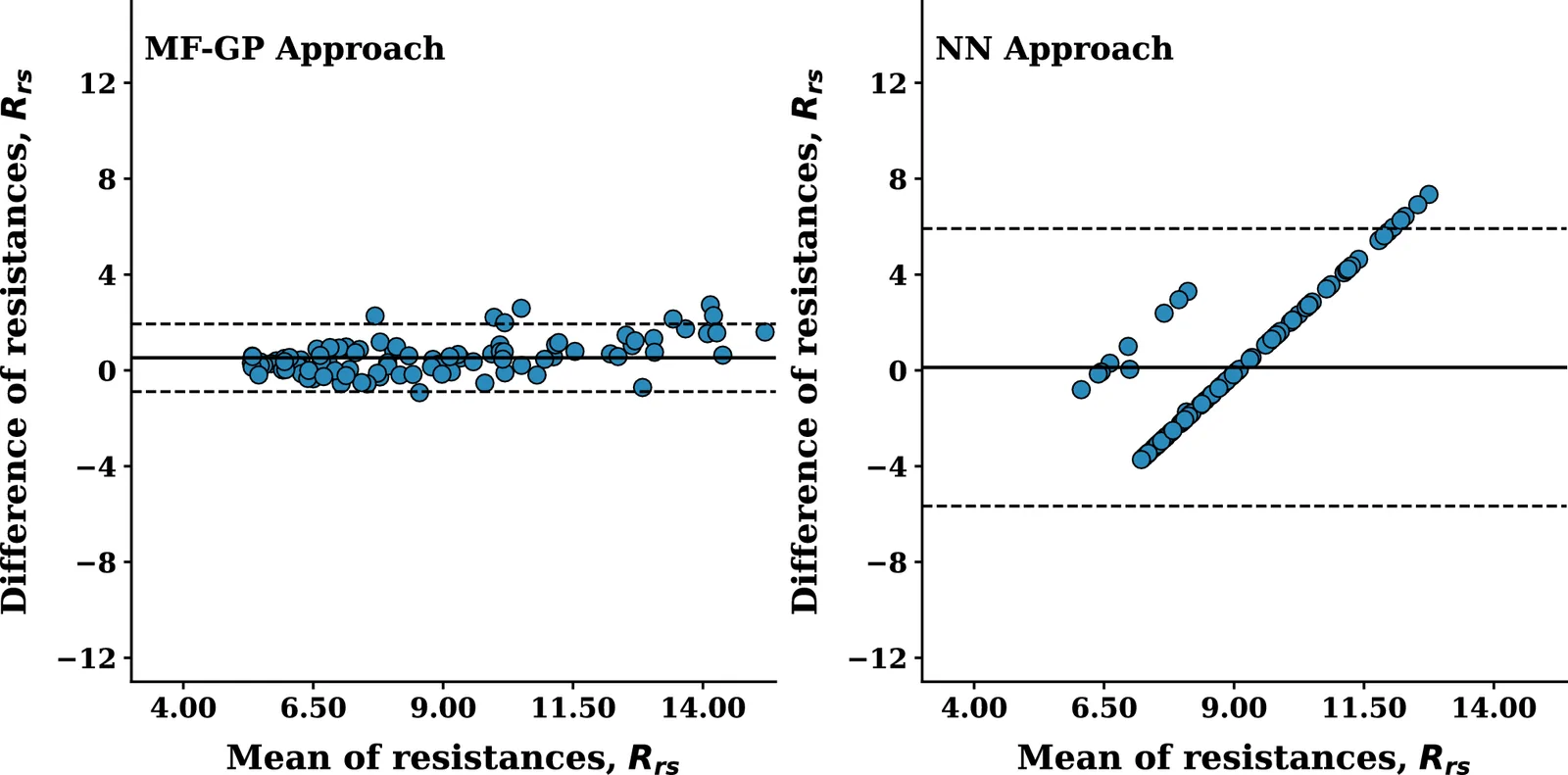
Bland–Altman plots for ${ R}_{\textrm{rs}}$: MF-GP approach (left) and NN approach (right). Solid line = mean bias; dashed lines = 95% limits of agreement

To further assess agreement between surrogate predictions and HF simulation outputs, Bland–Altman analyses (Bland and Altman 2010) were performed for both ${ C}_{\textrm{rs}}$ and ${ R}_{\textrm{rs}}$, as shown in Figs. 6 and 7. These plots display the difference between surrogate predictions and HF values against their mean, with the solid horizontal line representing the mean bias and dashed lines denoting the 95% limits of agreement.

For ${ C}_{\textrm{rs}}$, the MF-GP model exhibits near-zero mean bias with relatively narrow limits of agreement, and the residuals remain approximately randomly distributed around zero across the physiological range. This indicates minimal proportional bias and stable predictive performance. In contrast, the NN approach shows substantially wider scatter and broader limits of agreement, with increased variability at higher compliance values, indicating reduced robustness and poorer calibration relative to the MF-GP model. A similar trend is observed for ${ R}_{\textrm{rs}}$. The MF-GP model demonstrates relatively small prediction bias and narrow limits of agreement over the full resistance range, indicating strong consistency with HF simulations. In contrast, the NN model exhibits pronounced systematic bias and highly structured residual behavior, with prediction errors increasing monotonically with resistance magnitude. The near-linear error trend confirms that the NN surrogate fails to adequately resolve resistance dynamics under limited HF training data.

**Fig. 8:**
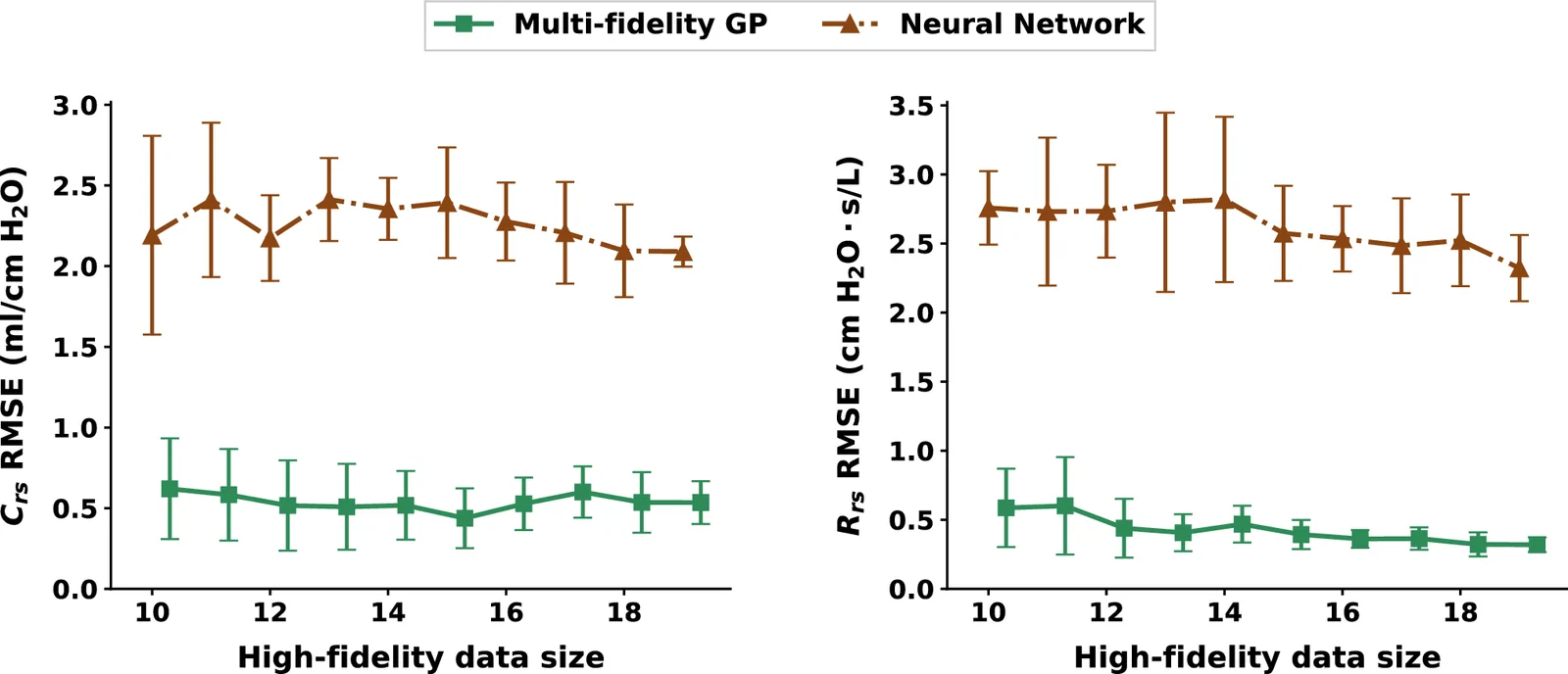
RMSE of ${ C}_{\textrm{rs}}$ (left) and ${ R}_{\textrm{rs}}$ (right) predictions as a function of HF training data size. Green squares: MF-GP; brown triangles: NN. Error bars denote standard deviation over repeated training realizations

To evaluate surrogate robustness under varying HF data availability, the root-mean-square error (RMSE) was computed for both ${ C}_{\textrm{rs}}$ and ${ R}_{\textrm{rs}}$ as a function of HF training dataset size (10–19 samples), as shown in Fig. 8. For ${ C}_{\textrm{rs}}$, the MF-GP model consistently maintains low prediction error across all training sizes, with RMSE remaining approximately within 0.45 mL/cmH$_{2}$O to 0.65 mL/cmH$_{2}$O. In contrast, the NN exhibits substantially larger errors, with RMSE values approximately between 2.1 mL/cmH$_2$O to 2.4 mL/cmH$_2$O, together with larger uncertainty across repeated training runs. For ${ R}_{\textrm{rs}}$, the MF-GP again demonstrates superior robustness, maintaining RMSE values below approximately 0.6 cmH$_2$Os/L across all HF dataset sizes. The NN model shows considerably larger prediction error (≈ 2.3 cmH$_{2}$Os/L to 2.8 cmH$_{2}$Os/L) and substantially higher variability, indicating unstable generalization performance in the sparse-data regime.

The superior performance of the MF-GP model is consistent with previous studies demonstrating that Gaussian process–based surrogate models are particularly effective for small-data scientific machine learning applications due to their nonparametric formulation and inherent uncertainty quantification capability (Kennedy and O’Hagan 2000; Williams and Rasmussen 2006). In contrast, neural networks generally require substantially larger training datasets to achieve comparable predictive robustness and stability (Goodfellow et al. 2016). Overall, these results demonstrate that the MF-GP surrogate substantially outperforms the NN approach for predicting both ${ C}_{\textrm{rs}}$ and ${ R}_{\textrm{rs}}$ within the limited HF training regime considered in this study. The MF-GP model exhibits stronger correlation with HF simulations, lower RMSE, narrower limits of agreement, and improved robustness across varying training dataset sizes. Therefore, within the scope of this study, the MF-GP surrogate is selected for subsequent sensitivity analysis and uncertainty quantification. Nevertheless, additional investigations using larger HF datasets and alternative neural network architectures remain necessary to evaluate the generality of these findings across broader respiratory-mechanics modeling scenarios.:contentReference[oaicite:0]index=0

#### Validation and relevance of the MF-GP surrogate

The MF-GP–predicted lung mechanics (Table 7) fall within established physiological ranges for juvenile porcine subjects (Table 5). Specifically, the respiratory system compliance ${C}_{\text {rs}}$ = 6.52mL/cmH2O lies within the literature range 5.68 mL/cmH2O to 8.20 mL/cmH2O, and the respiratory system resistance ${R}_{\text {rs}}$ = 7.54 cmH2Os/L lies within 5.7 cmH2Os/L to 12.1 cmH2Os/L.

**Table 7:** Predicted lung mechanics for the ML-based approach

Parameter	ML-based approach
Baseline physical model parameter Values	$c = 0.535~\textrm{kPa}$, β=1.075, $c_{1} = 0.2782~\textrm{kPa}$, $c_{3} = 5.766\times 10^{-3}~\textrm{kPa}$, $k = 3\times 10^4~\mathrm {mm^2/(kPa\cdot s)}$, $K_{\textrm{cw}} = 0.08~\mathrm {kPa/mm}$
Respiratory compliance ($\textit{C}_{\text {rs}}$)	$6.52~\mathrm {mL/cmH_2O}$
Respiratory resistance ($\textit{R}_{\text {rs}}$)	$7.54~\mathrm {cmH_2O\cdot s/L}$
Computation time	$0.0124~\textrm{s}$

Moreover, the MF-GP surrogate demonstrates strong agreement with both the high-fidelity (HF) and low-fidelity (LF) poroelastic FE simulations (Table 6) while maintaining substantially lower computational cost. For the baseline parameter set, the MF-GP predicts respiratory compliance and resistance values of ${C}_{\textrm{rs}}=6.52\textrm{mL}/\textrm{cmH}_{2}O$ and ${ R}_{\textrm{rs}}=7.54 \textrm{cmH}_{2}\textrm{Os}/\textrm{L}$, compared with HF values of $6.35\,\textrm{mL}/\textrm{cmH}_{2}\textrm{O}$ and $8.78 \textrm{cmH}_{2}\textrm{Os}/\textrm{L},$, respectively. This corresponds to absolute errors of approximately $0.17\,\textrm{mL}/\textrm{cmH}_{2}\textrm{O}$ ($\sim 2.7\%$) for ${ C}_{\textrm{rs}}$ and $1.24 \textrm{cmH}_{2}\textrm{Os}/\textrm{L}$ ($\sim 14.1\%$) for ${ R}_{\textrm{rs}}$ relative to the HF reference solution.

In addition, the LF FE model predicts ${ C}_{\textrm{rs}}=6.12\,\textrm{mL}/\textrm{cmH}_{2}\textrm{O}$ and ${ R}_{\textrm{rs}}=8.13 \textrm{cmH}_{2}\textrm{Os}/\textrm{L}$, indicating that the MF-GP surrogate preserves the dominant global respiratory mechanics captured by both FE formulations. While the HF simulation requires approximately 3642.9 s and the LF simulation requires 177 s, the MF-GP inference is completed in only 0.0124 s. This corresponds to an acceleration factor of approximately $2.9\times 10^{5}$ relative to the HF simulation and approximately $1.4\times 10^{4}$ relative to the LF model. This combination of strong physiological agreement, predictive accuracy, and several orders-of-magnitude computational acceleration demonstrates that the MF-GP surrogate provides an efficient framework for rapid respiratory-mechanics estimation, uncertainty-aware prediction, and inverse parameter identification.

### Global sensitivity analysis

We performed a variance-based global sensitivity analysis using Sobol’s indices to quantify the relative influence of key poroelastic parameters on respiratory mechanics. The analysis was conducted over physiologically plausible parameter ranges selected from literature and prior calibration studies, ensuring consistency with observed porcine lung behavior. All parameters were assumed to follow independent uniform distributions within their respective ranges, reflecting non-informative priors over feasible physiological bounds.

**Fig. 9:**
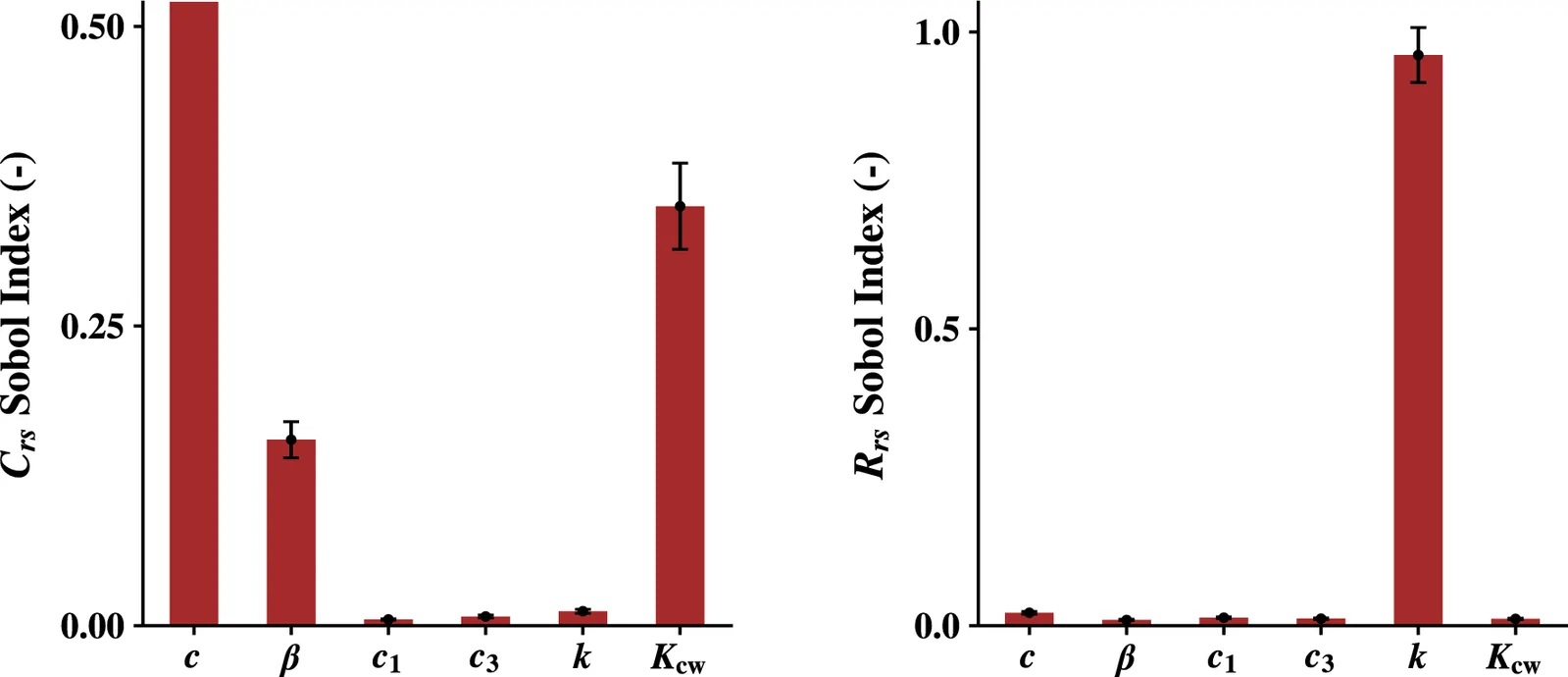
First-order Sobol indices for ${ C}_{\text {rs}}$ (left) and ${ R}_{\text {rs}}$ (right), quantifying the main effect of each constitutive parameter. Error bars represent 95% confidence intervals estimated via Monte Carlo sampling

Figure 9 displays the first-order Sobol indices for six input parameters—elastic stiffness (*c*), nonlinear coefficient (β), hyperelastic constants ($c_1, c_3$), permeability (*k*), and chest-wall stiffness ($K_{\textrm{cw}}$)—with respect to respiratory compliance (${ C}_{\text {rs}}$) and respiratory resistance (${ R}_{\text {rs}}$). Error bars denote 95% confidence intervals computed via bootstrap resampling over 100 Monte Carlo replicates.

As shown in the left panel, respiratory compliance (${ C}_{\text {rs}}$) is primarily influenced by elastic stiffness (*c*) and chest-wall stiffness ($K_{\textrm{cw}}$), which together account for the majority of the output variance. The nonlinear coefficient (β) contributes modestly, while hyperelastic constants ($c_1$, $c_3$) and permeability (*k*) exhibit negligible first-order effects within the considered parameter space. In contrast, respiratory resistance (${ R}_{\text {rs}}$) is predominantly governed by permeability (*k*), which accounts for the majority of the variance, while all other parameters contribute minimally.

**Fig. 10:**
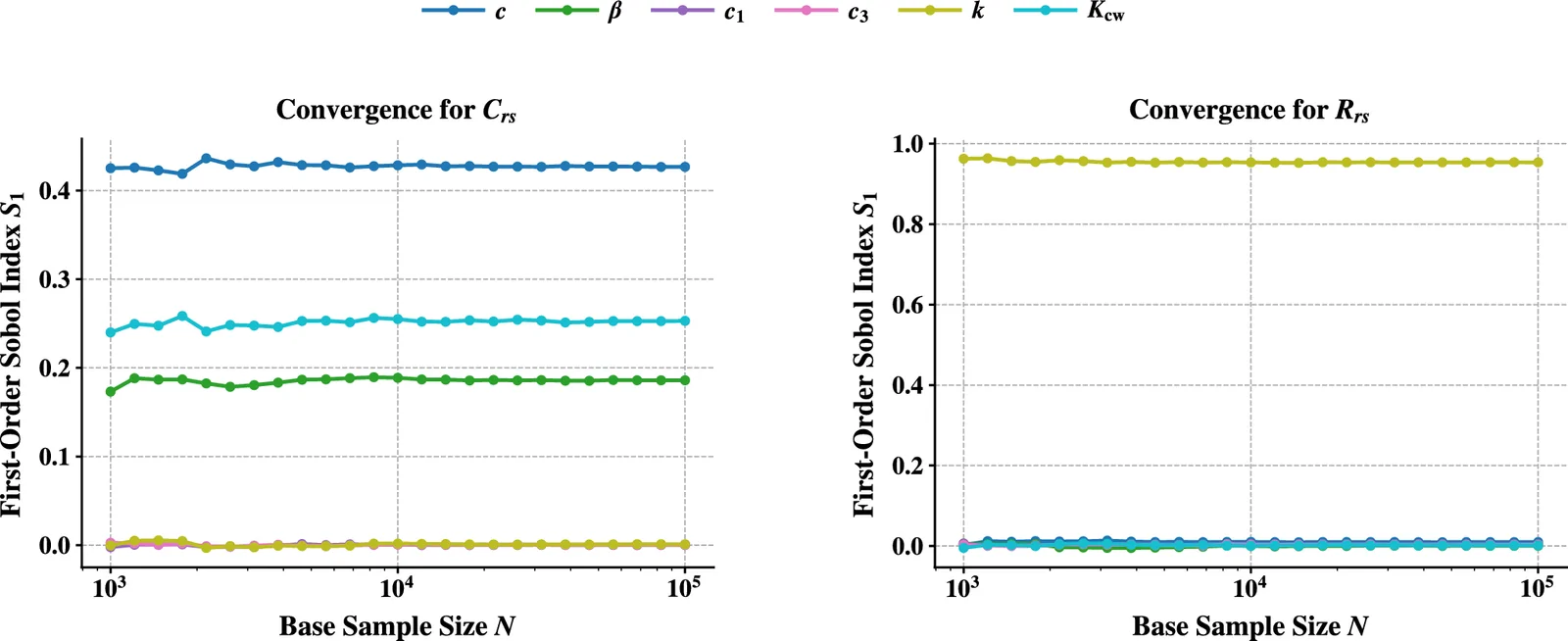
Sobol index convergence analysis

Figure 10 illustrates convergence of the first-order Sobol indices as a function of base sample size *N* ($10^3 \le N \le 10^5$). For ${ C}_{\text {rs}}$, indices stabilize beyond $N \approx 10^4$, with *c* and $K_{\textrm{cw}}$ emerging as dominant contributors, while remaining parameters retain low influence. For ${ R}_{\text {rs}}$, *k* consistently dominates across all sample sizes, indicating strong robustness of the ranking with respect to sampling density.

These results highlight a strong separation between compliance- and resistance-dominated mechanisms within the model. However, it is important to note that Sobol’s indices are conditional on the selected parameter ranges and assumed distributions. Therefore, while parameters such as $c_1$, $c_3$, and β exhibit negligible influence in the present study, this should not be interpreted as general physiological insignificance, but rather as a statement valid within the specified modeling and uncertainty framework.

From a modeling perspective, these findings support reduced-order representations under the present parameterization, where dominant physical model parameters (*c*, $K_{\textrm{cw}}$, and *k*) primarily govern system variability. However, such simplifications are regime-dependent and should be revisited when parameter ranges, boundary conditions, or patient-specific physiology change. Clinically, the strong sensitivity of ${ R}_{\text {rs}}$ to permeability (*k*) highlights its importance in characterizing fluid transport behavior, while compliance is governed by coupled solid and boundary mechanics.

#### One-at-a-time parameter sweeps

We conducted one-at-a-time (OAT) parameter sweeps over physiologically plausible ranges while holding all remaining physical model parameters fixed at their baseline values. The objective was to complement the Sobol sensitivity analysis and directly visualize the functional dependence of respiratory compliance (${ C}_{\textrm{rs}}$) and respiratory resistance (${ R}_{\textrm{rs}}$) on individual poroelastic parameters.

Whereas Sobol indices quantify global parameter importance through variance decomposition, they do not explicitly reveal the shape of the input–output relationship. The OAT sweeps therefore provide additional mechanistic insight by identifying monotonic, nonlinear, or weakly varying parameter responses and by qualitatively validating the weak interaction effects inferred from the Sobol analysis.

**Fig. 11:**
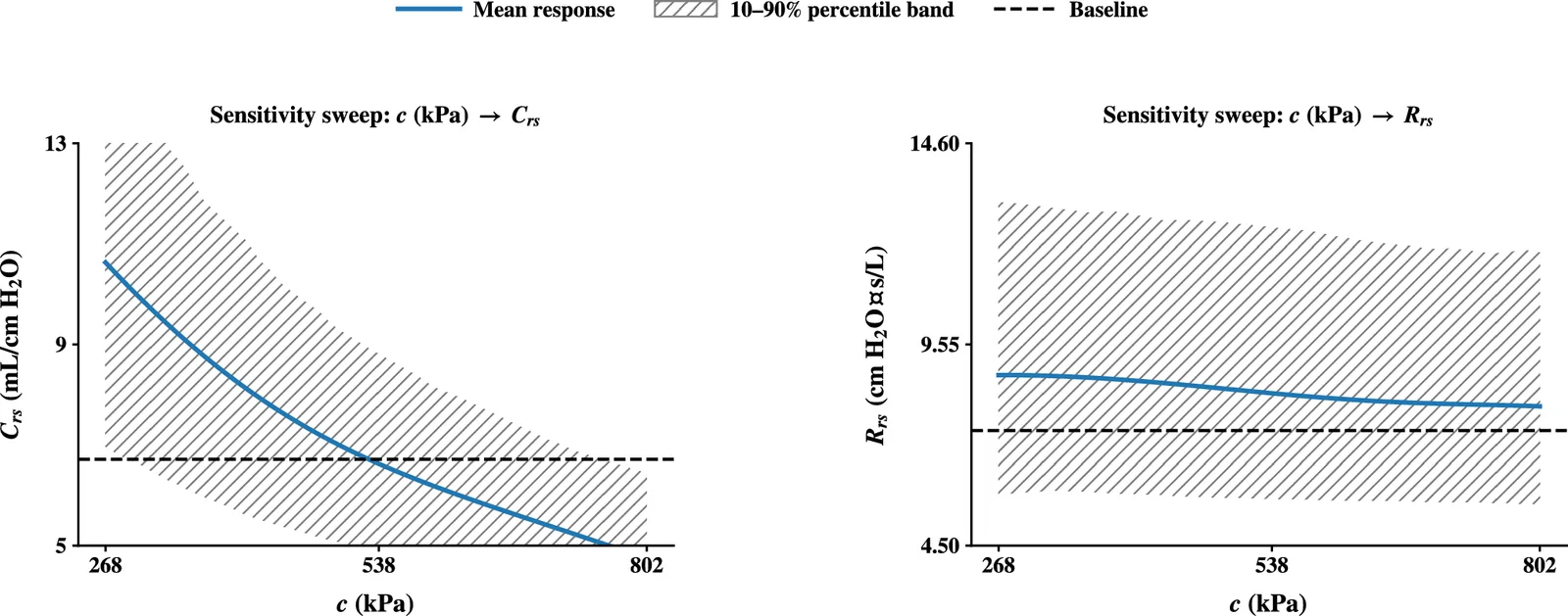
Sensitivity sweep of parameter *c*: effect on ${ C}_{\textrm{rs}}$ (left) and ${ R}_{\textrm{rs}}$ (right). The solid blue line denotes the mean surrogate prediction, the shaded region represents the 10–90% prediction interval, and the dashed line indicates the baseline value

**Fig. 12:**
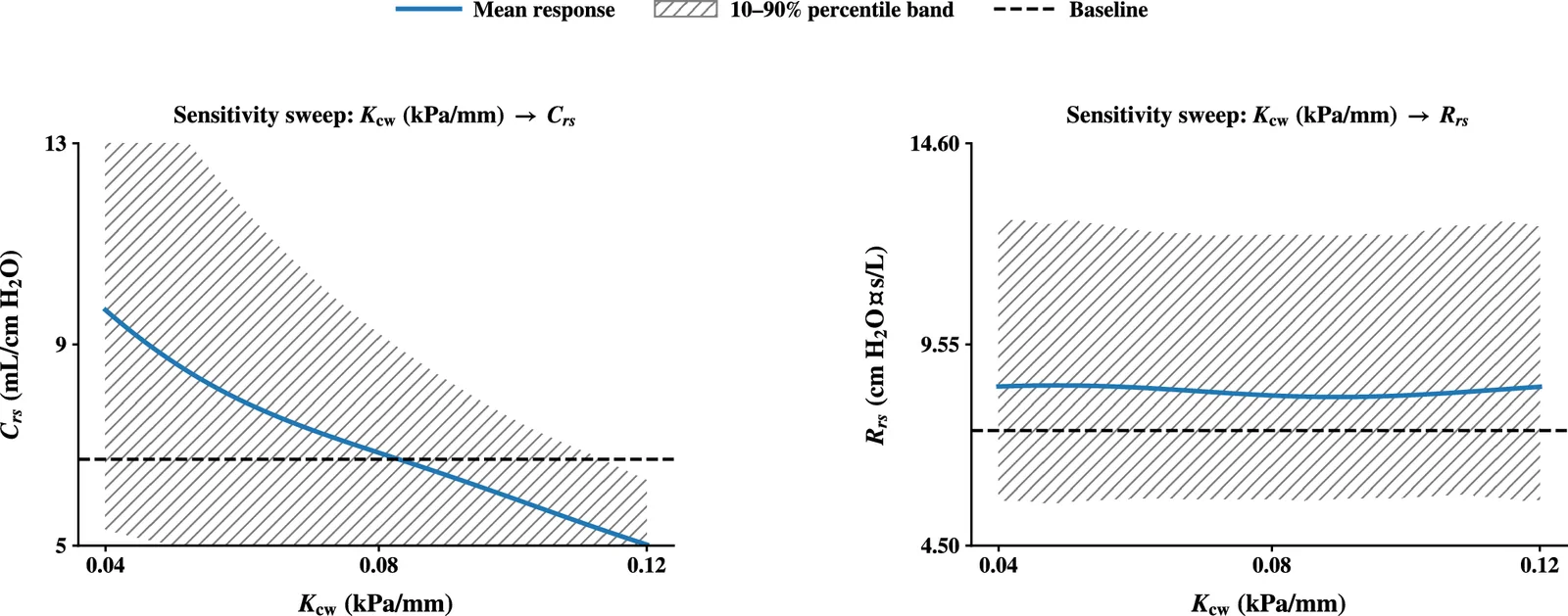
Sensitivity sweep of parameter $K_{\textrm{cw}}$: effect on ${ C}_{\textrm{rs}}$ (left) and ${ R}_{\textrm{rs}}$ (right)

The resulting trends are shown in Figs. 11, 12 and are fully consistent with the variance-based sensitivity results.

Figures 11 and 12 demonstrate that both *c* and $K_{\textrm{cw}}$ strongly influence ${ C}_{\textrm{rs}}$. Increasing either parameter produces a pronounced monotonic decrease in compliance, with ${ C}_{\textrm{rs}}$ decreasing from approximately 10 mL/cmH$_{2}$O to 5 mL/cmH$_{2}$O across the investigated ranges. The nonlinear decay indicates strong sensitivity of global compliance to tissue stiffness and chest-wall coupling. In contrast, both parameters produce only minor variations in ${ R}_{\textrm{rs}}$, confirming their dominant role in elastic rather than resistive respiratory mechanics.

**Fig. 13:**
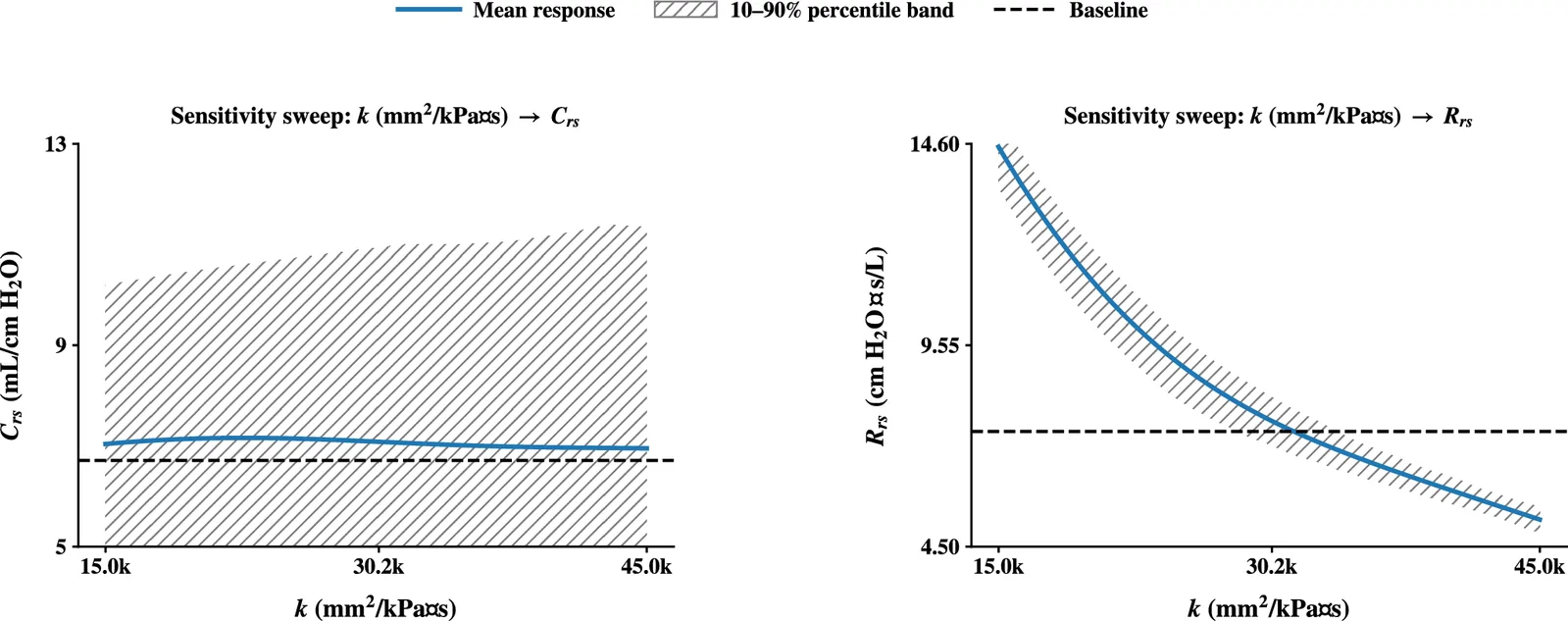
Sensitivity sweep of parameter *k*: effect on ${ C}_{\textrm{rs}}$ (left) and ${ R}_{\textrm{rs}}$ (right)

The permeability parameter *k* exhibits the opposite behavior (Fig. 13). Increasing permeability produces a strong nonlinear reduction in ${ R}_{\textrm{rs}}$, with resistance decreasing from approximately 14.5 cmH$_{2}$Os/L to 5 cmH$_{2}$Os/L over the tested range. This behavior is consistent with Darcy-type transport, where larger permeability facilitates interstitial fluid transport and reduces effective resistive loading. In contrast, the effect of *k* on ${ C}_{\textrm{rs}}$ remains minimal, with only small fluctuations around the baseline compliance value. These results confirm the dominant role of permeability in governing resistive rather than elastic respiratory dynamics.

**Fig. 14:**
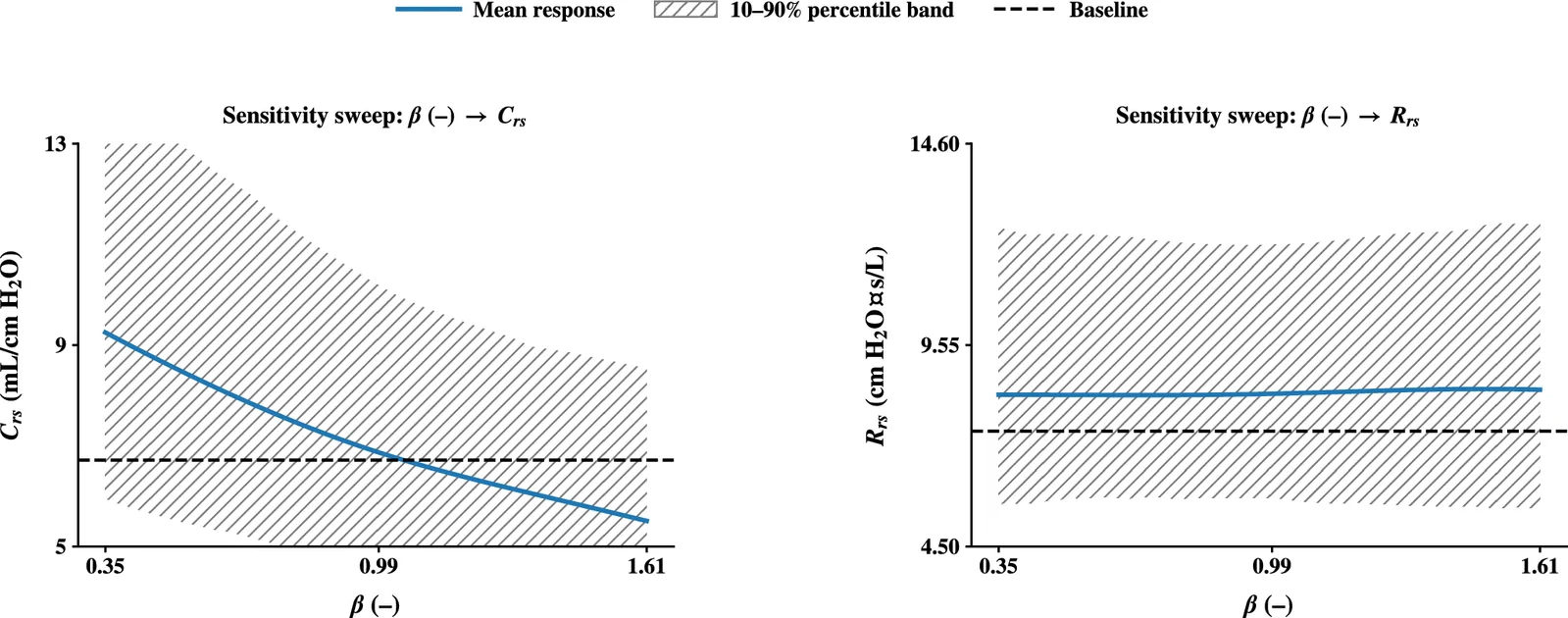
Sensitivity sweep of parameter β: effect on ${ C}_{\textrm{rs}}$ (left) and ${ R}_{\textrm{rs}}$ (right)

**Fig. 15:**
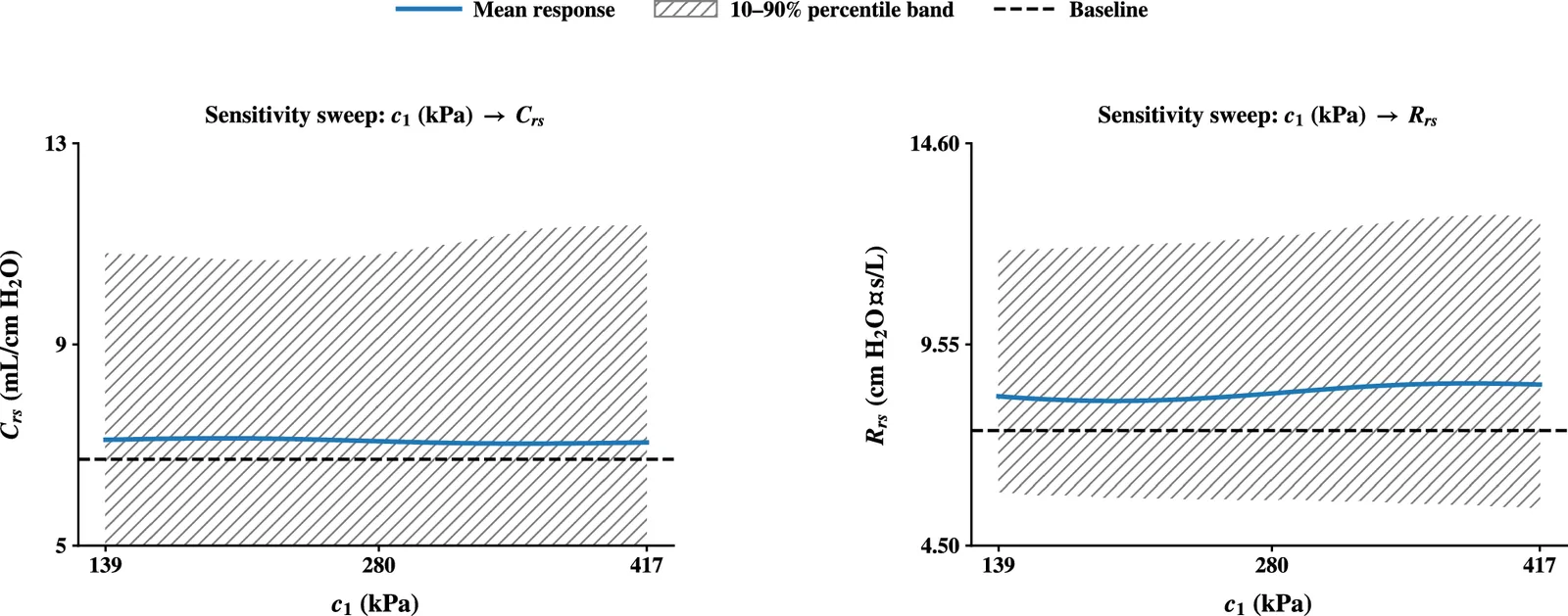
Sensitivity sweep of parameter $c_1$: effect on ${ C}_{\textrm{rs}}$ (left) and ${ R}_{\textrm{rs}}$ (right)

**Fig. 16:**
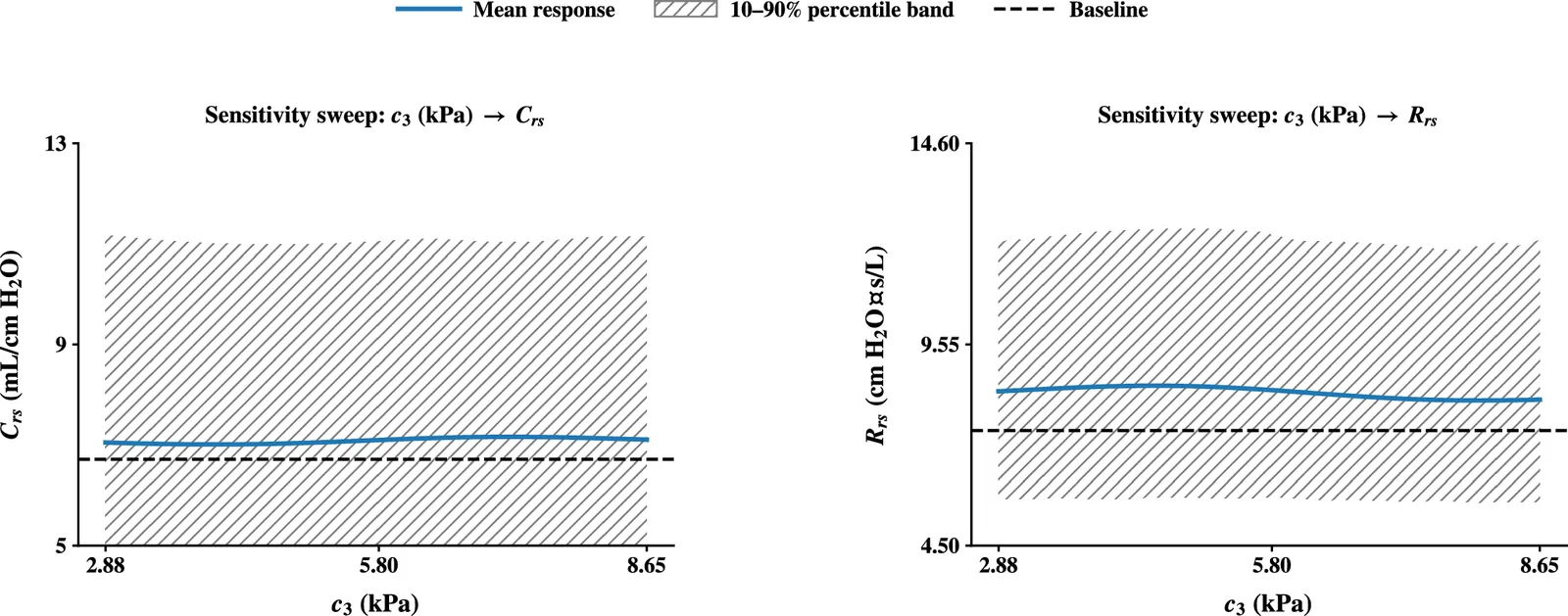
Sensitivity sweep of parameter $c_3$: effect on ${ C}_{\textrm{rs}}$ (left) and ${ R}_{\textrm{rs}}$ (right)

Figures 14, 15, 16 show that variations in β, $c_1$, and $c_3$ produce comparatively weak responses in both ${ C}_{\textrm{rs}}$ and ${ R}_{\textrm{rs}}$. Although small monotonic trends are observable for β, the overall variation remains limited relative to the dominant parameters *c*, $K_{\textrm{cw}}$, and *k*. Similarly, $c_1$ and $c_3$ generate only minor deviations from the baseline response, with nearly flat mean-response curves and broad uncertainty bands relative to the magnitude of the response variation. These results confirm the relatively weak influence of these constitutive parameters on global respiratory mechanics within the investigated parameter ranges.

Overall, the OAT sweeps reveal a clear decoupling between the dominant elastic and transport mechanisms governing respiratory mechanics. Compliance is primarily controlled by the tissue stiffness parameter *c* and the chest-wall coupling parameter $K_{\textrm{cw}}$, whereas resistance is dominated by permeability *k*. The remaining constitutive parameters (β, $c_1$, and $c_3$) exert comparatively weak influence on the global outputs. The largely monotonic response trends and the absence of strongly nonlinear coupled behavior further support the approximately additive structure suggested by the Sobol sensitivity analysis.

These findings also explain the strong predictive performance of the MF-GP surrogate model. Because the dominant respiratory responses are governed by a small subset of parameters with smooth low-dimensional trends, the surrogate can efficiently learn the mapping between physical model parameters and respiratory outputs. Consequently, the results support a reduced-order calibration strategy in which *c*, $K_{\textrm{cw}}$, and *k* are prioritized during inverse parameter estimation, while β, $c_1$, and $c_3$ may be fixed near their baseline values without substantial loss of predictive accuracy.

### Uncertainty quantification and parameter-specific variability

The impact of parametric uncertainty on respiratory compliance (${ C}_{\textrm{rs}}$) and respiratory resistance (${ R}_{\textrm{rs}}$) was investigated using forward uncertainty quantification based on the MF-GP surrogate model. Each physical model parameter was independently perturbed within $\pm 10\%$, $\pm 25\%$, and $\pm 50\%$ of its baseline value while the remaining parameters were fixed. In addition, a combined uncertainty scenario was considered in which all six physical model parameters were simultaneously perturbed over the same uncertainty ranges. For each case, 10,000 MF-GP realizations were generated and probability density functions (PDFs) were estimated from the resulting output distributions.

**Fig. 17:**
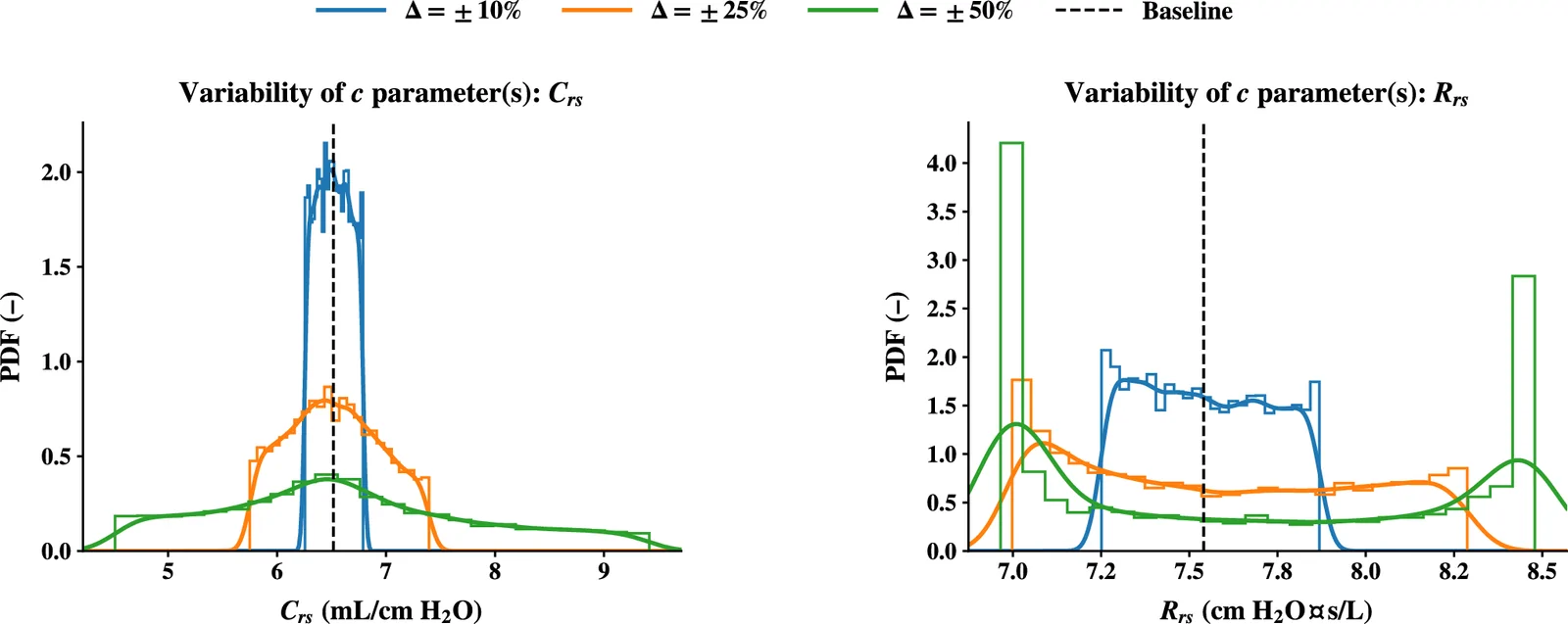
Propagation of uncertainty in parameter *c* on ${ C}_{\textrm{rs}}$ (left) and ${ R}_{\textrm{rs}}$ (right). The blue, orange, and green curves correspond to $\pm 10\%$, $\pm 25\%$, and $\pm 50\%$ parameter perturbations, respectively. The dashed vertical line denotes the baseline prediction

The uncertainty distributions reveal a strong mechanistic separation between the parameters governing compliance and those governing resistance. In particular, uncertainty in the tissue stiffness parameter *c* produces substantial broadening of the ${ C}_{\textrm{rs}}$ distributions while only minimally affecting ${ R}_{\textrm{rs}}$ (Fig. 17). As the perturbation level increases from $\pm 10\%$ to $\pm 50\%$, the compliance distributions broaden significantly and become increasingly skewed toward larger compliance values, indicating strong nonlinear sensitivity of global lung compliance to tissue elasticity. In contrast, the associated resistance distributions remain comparatively narrow and centered near the baseline prediction, confirming the weak influence of *c* on resistive mechanics.

**Fig. 18:**
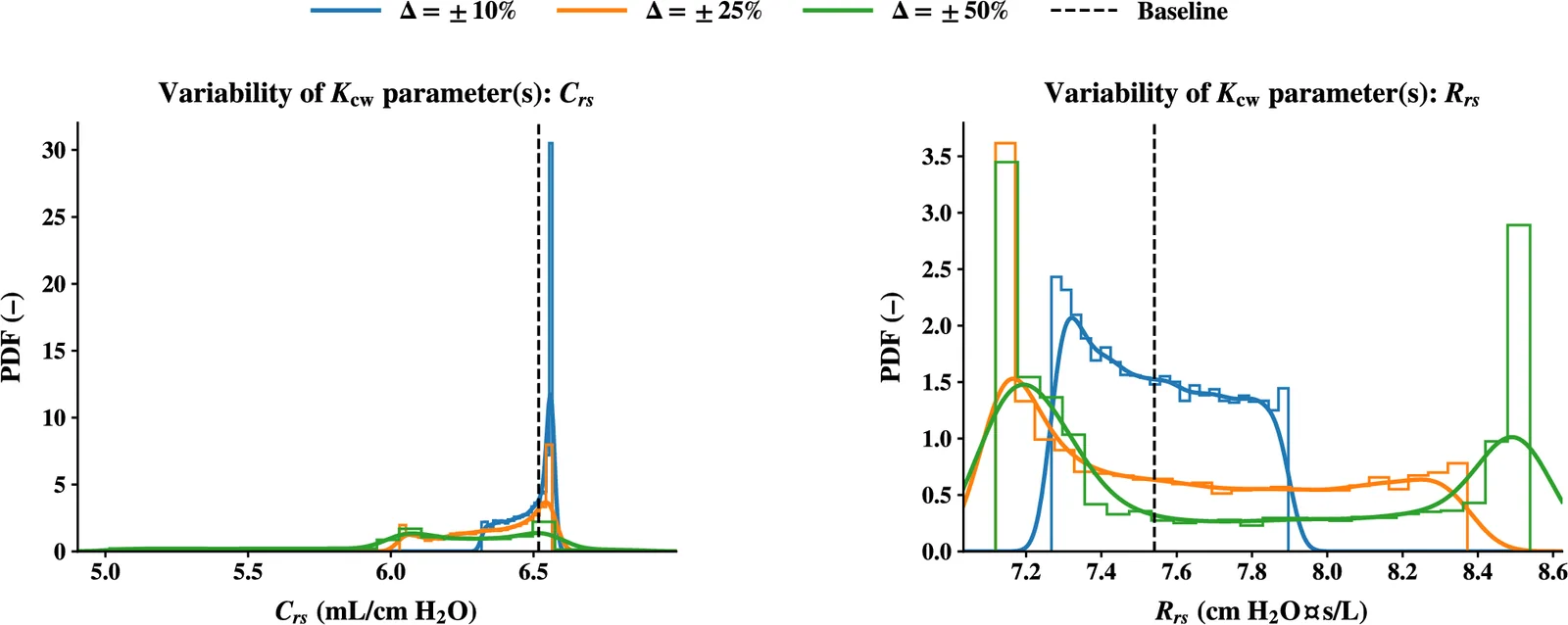
Propagation of uncertainty in parameter $K_{\textrm{cw}}$ on ${ C}_{\textrm{rs}}$ (left) and ${ R}_{\textrm{rs}}$ (right)

A similarly strong effect is observed for the chest-wall coupling parameter $K_{\textrm{cw}}$ (Fig. 18). Increasing uncertainty in $K_{\textrm{cw}}$ generates pronounced dispersion in ${ C}_{\textrm{rs}}$, with the distributions becoming highly concentrated around the baseline value at small perturbation levels and substantially broader at $\pm 50\%$ variation. However, the corresponding ${ R}_{\textrm{rs}}$ distributions remain relatively stable, further confirming that chest-wall coupling primarily governs elastic rather than resistive respiratory behavior.

**Fig. 19:**
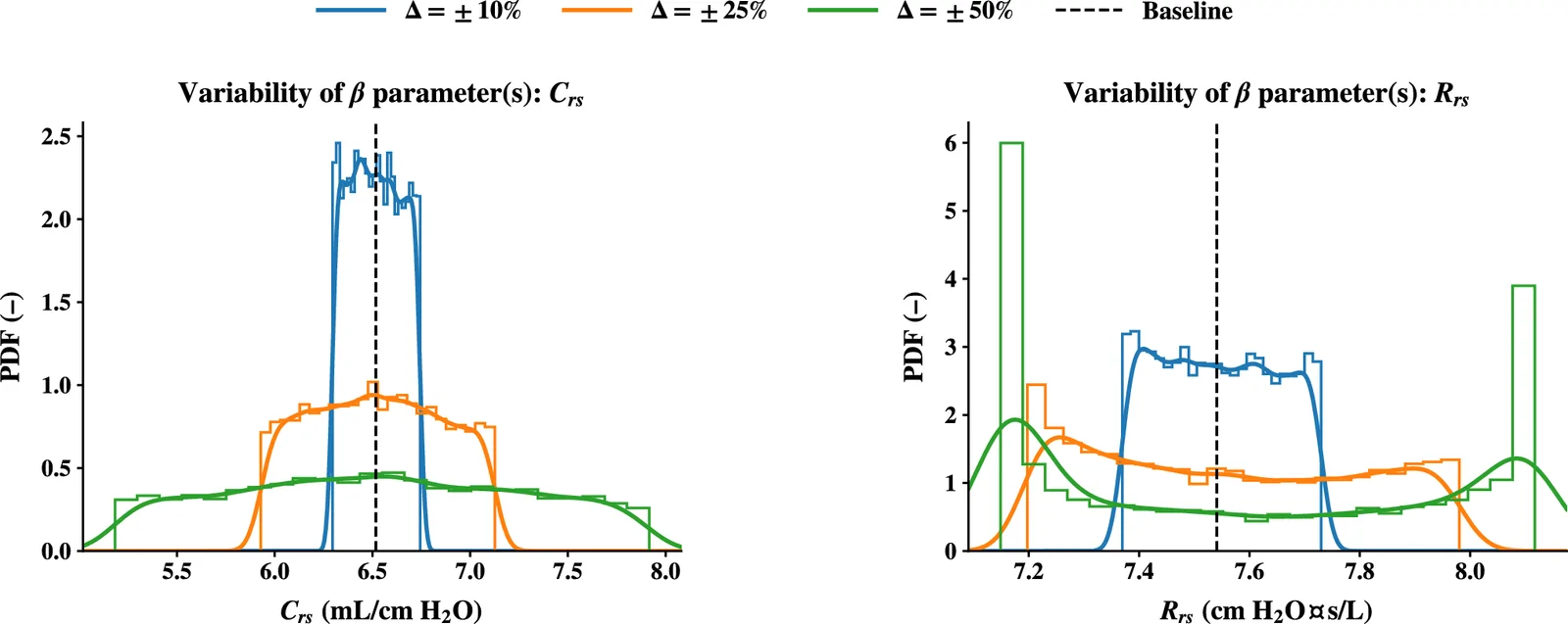
Propagation of uncertainty in parameter β on ${ C}_{\textrm{rs}}$ (left) and ${ R}_{\textrm{rs}}$ (right)

The nonlinear constitutive parameter β exhibits comparatively weaker effects on both outputs (Fig. 19). Although increasing perturbation levels broaden the ${ C}_{\textrm{rs}}$ distributions, the variability remains moderate relative to that induced by *c* or $K_{\textrm{cw}}$. The resulting ${ R}_{\textrm{rs}}$ distributions show only small shifts around the baseline prediction, indicating limited influence on global respiratory resistance.

**Fig. 20:**
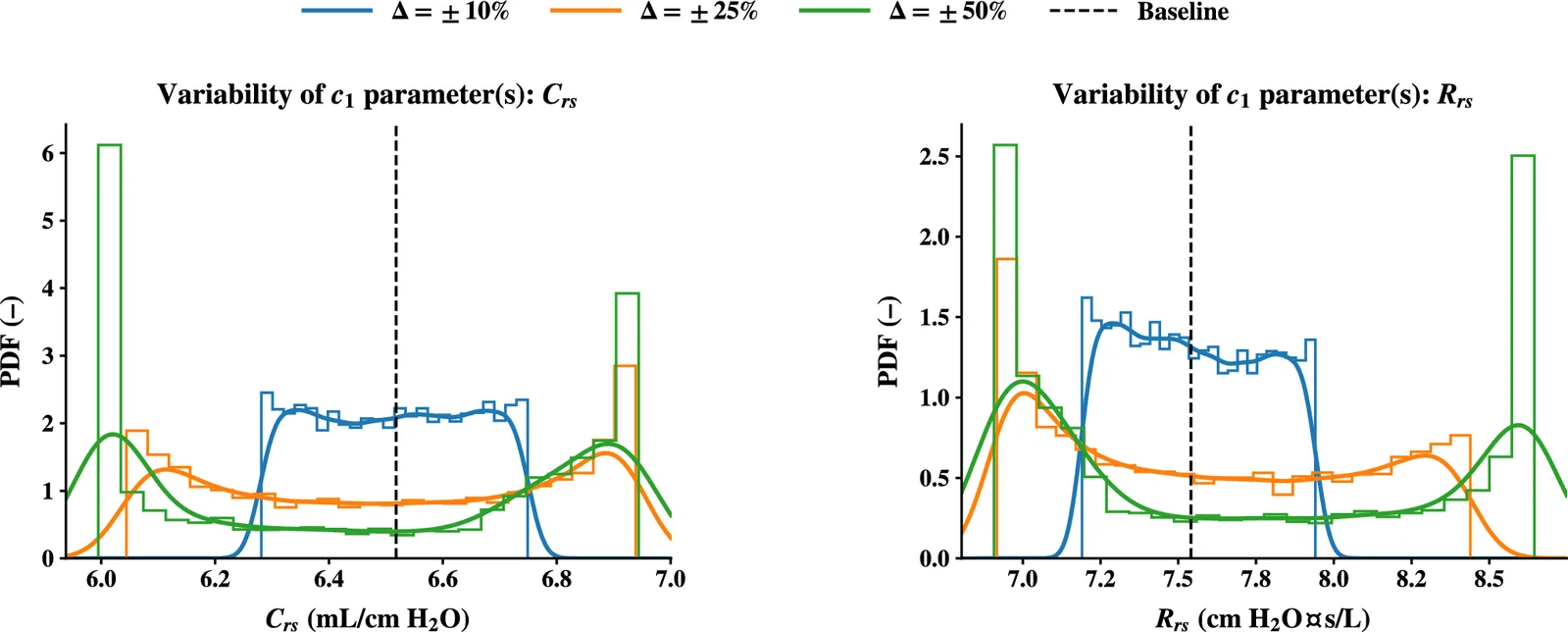
Propagation of uncertainty in parameter $c_1$ on ${ C}_{\textrm{rs}}$ (left) and ${ R}_{\textrm{rs}}$ (right)

**Fig. 21:**
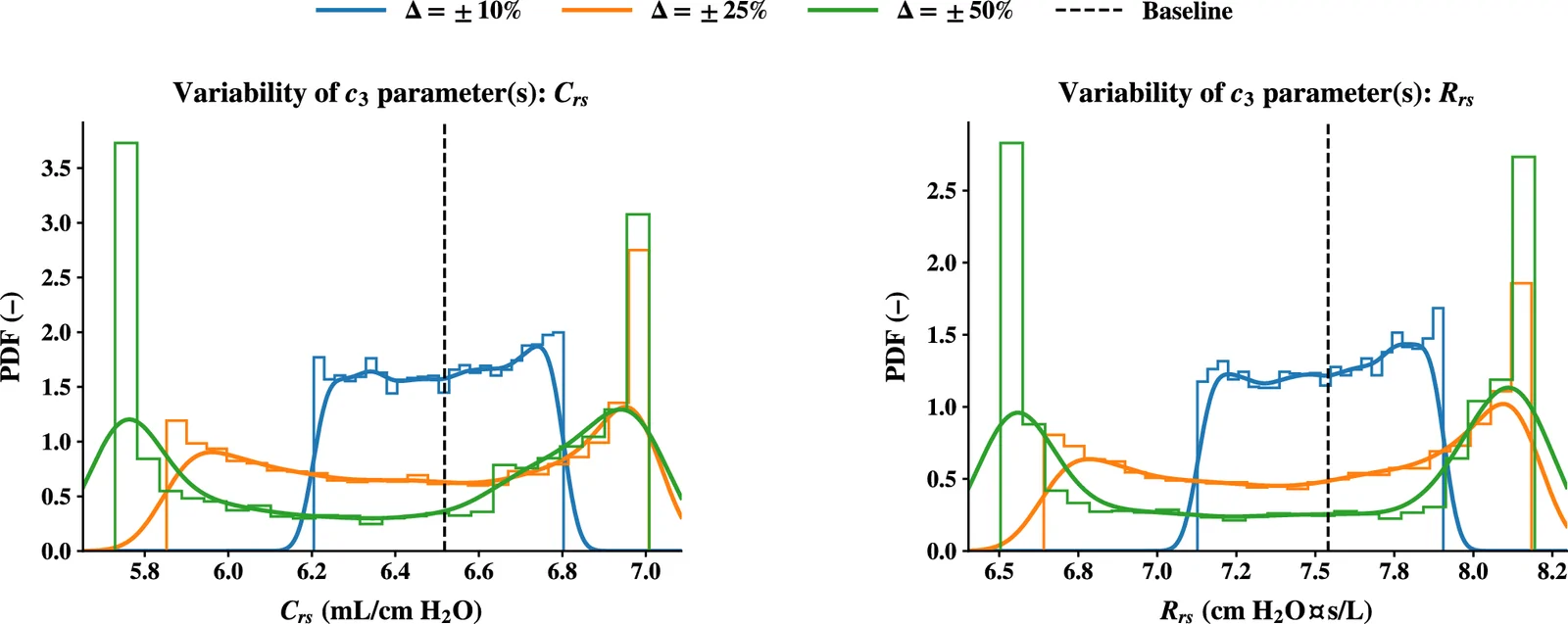
Propagation of uncertainty in parameter $c_3$ on ${ C}_{\textrm{rs}}$ (left) and ${ R}_{\textrm{rs}}$ (right)

The fiber-related constitutive parameters $c_1$ and $c_3$ produce relatively small variations in both respiratory outputs (Figs. 20 and 21). Even under $\pm 50\%$ perturbations, the resulting distributions remain comparatively narrow and centered near the baseline predictions, demonstrating that these parameters exert only limited influence on global respiratory mechanics within the investigated parameter ranges.

**Fig. 22:**
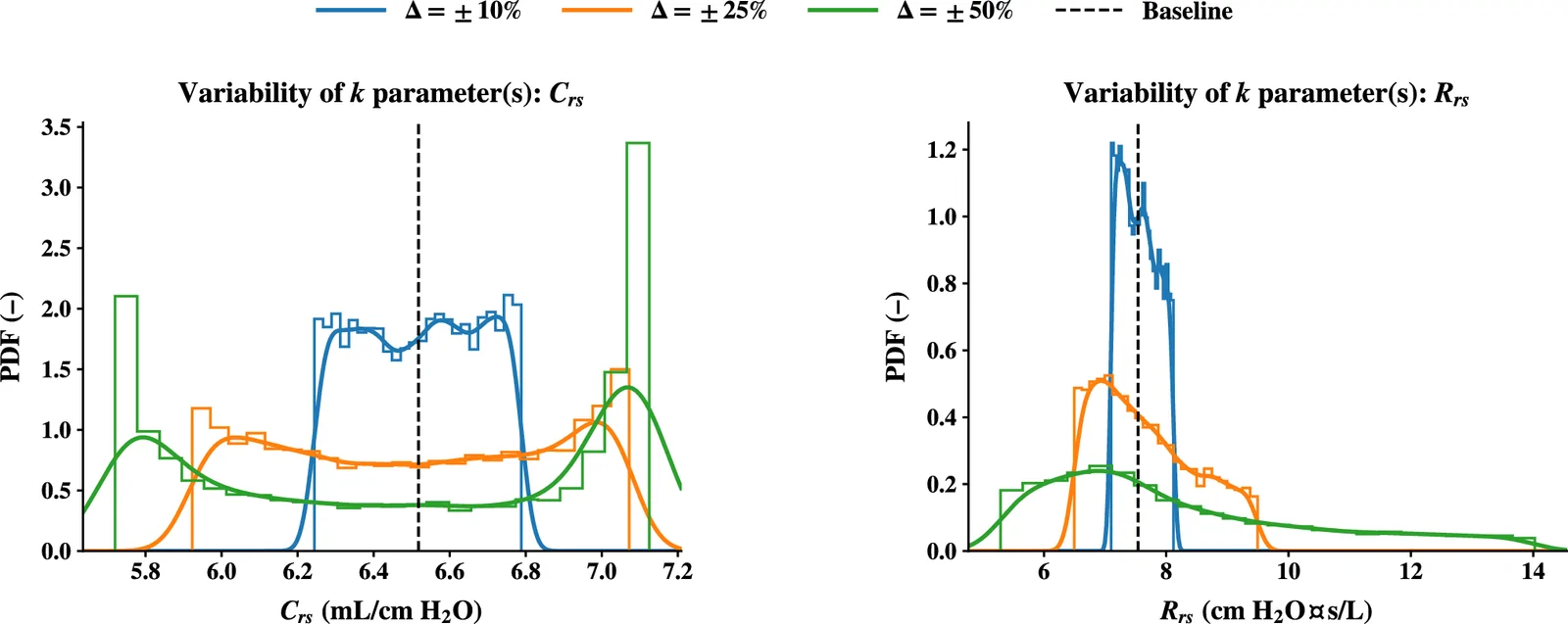
Propagation of uncertainty in parameter *k* on ${ C}_{\textrm{rs}}$ (left) and ${ R}_{\textrm{rs}}$ (right)

Permeability (*k*) exhibits the opposite behavior (Fig. 22). Its effect on ${ C}_{\textrm{rs}}$ remains modest, with only moderate broadening of the compliance distributions even at $\pm 50\%$ perturbation levels. In contrast, uncertainty in *k* produces substantial widening and pronounced right-skewness in the ${ R}_{\textrm{rs}}$ distributions. As the perturbation range increases, the resistance distributions broaden dramatically and shift toward larger values, confirming that interstitial permeability is the dominant determinant of resistive respiratory behavior within the poroelastic framework.

**Fig. 23:**
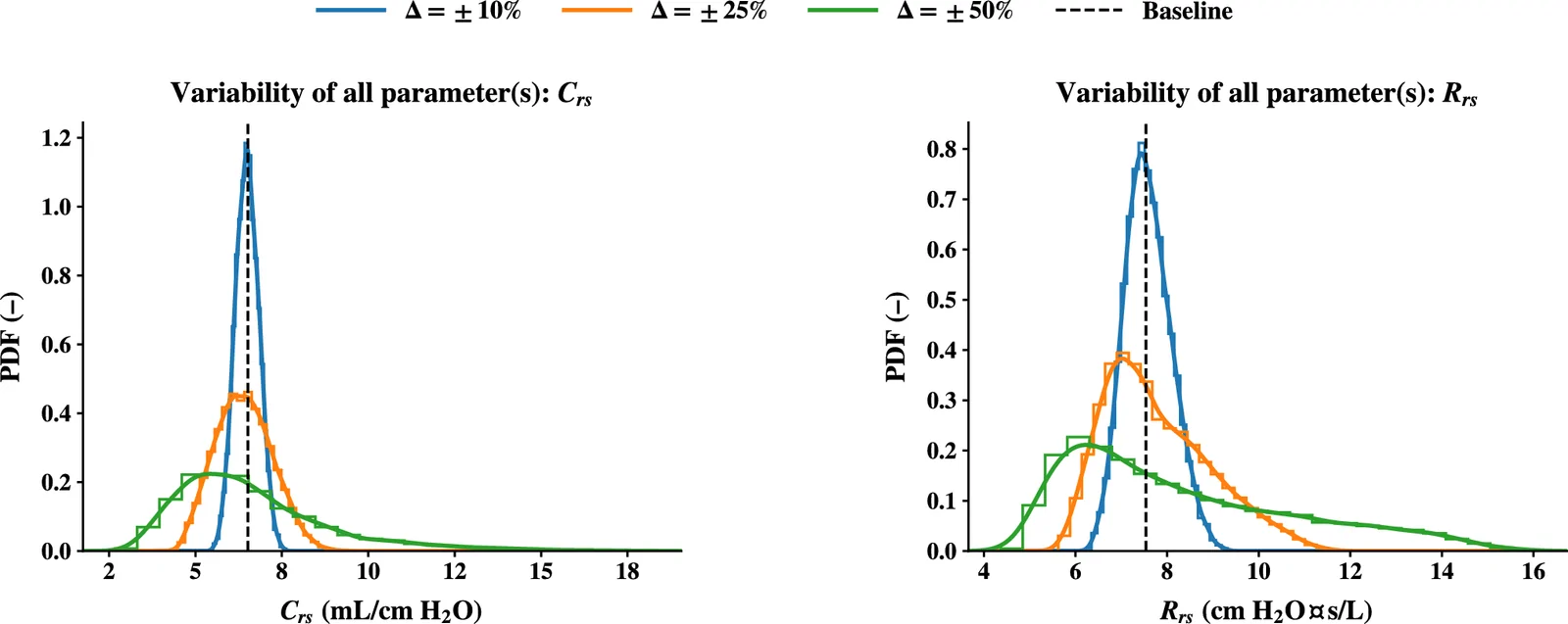
Combined propagation of uncertainty from all six physical model parameters on ${ C}_{\textrm{rs}}$ (left) and ${ R}_{\textrm{rs}}$ (right)

When all six physical model parameters are simultaneously perturbed, the resulting output distributions reflect the combined influence of the dominant elastic and transport mechanisms (Fig. 23). The ${ C}_{\textrm{rs}}$ distributions broaden progressively with increasing uncertainty levels and develop noticeable right-skewness, primarily due to the combined effects of *c* and $K_{\textrm{cw}}$. Meanwhile, the ${ R}_{\textrm{rs}}$ distributions closely resemble those obtained under permeability perturbations alone, confirming that *k* remains the principal driver of resistance variability even in the presence of concurrent uncertainty in the elastic constitutive parameters.

Overall, the uncertainty analysis demonstrates a strong decoupling between the physical mechanisms governing compliance and resistance. Respiratory compliance is primarily controlled by the elastic tissue stiffness parameter *c* and the chest-wall coupling parameter $K_{\textrm{cw}}$, whereas respiratory resistance is overwhelmingly dominated by permeability *k*. The remaining constitutive parameters (β, $c_1$, and $c_3$) contribute comparatively little to output variability. This separation substantially improves parameter identifiability and supports reduced-order calibration strategies focusing primarily on *c*, $K_{\textrm{cw}}$, and *k*.

The MF-GP surrogate accurately reproduces these complex uncertainty distributions across all perturbation scenarios, confirming its suitability for efficient uncertainty-aware prediction, sensitivity analysis, inverse parameter estimation, and future real-time respiratory mechanics applications.

## Discussion

This study introduces a species-specific multi-fidelity Gaussian process (MF-GP) framework for uncertainty-aware characterization of global respiratory mechanics in porcine lungs. A key result is that the proposed approach achieves accurate prediction of respiratory compliance (${ C}_{rs}$) and resistance (${ R}_{rs}$) with errors below 5% relative to high-fidelity (HF) poroelastic finite-element (FE) simulations, while delivering computational speedups exceeding five orders of magnitude. This establishes a practical pathway for overcoming the prohibitive cost of HF poroelastic models in large-scale and time-sensitive applications. Rather than a fully validated clinical tool, the framework should be interpreted as a proof-of-concept surrogate modeling pipeline demonstrating the feasibility of combining HF simulations, low-fidelity (LF) models, and machine learning for efficient exploration of respiratory mechanics.

By integrating CT-derived poroelastic FE simulations with machine learning and uncertainty quantification, the framework balances physiological realism with computational efficiency. HF models resolve nonlinear tissue deformation and airflow at the parenchymal scale, while LF models enable efficient sampling of a physiologically relevant parameter space. Their fusion within the MF-GP surrogate enables accurate and uncertainty-aware inference of global respiratory mechanics at a fraction of the computational cost of full FE simulations.

A central contribution of this work is the insight it provides a systematic framework for estimating the respiratory parameters in lung mechanics. Global Sobol sensitivity analysis reveals a clear mechanistic separation of parameter influence: compliance is primarily governed by elastic stiffness (c) and chest-wall coupling ($K_{\textrm{cw}}$), whereas resistance is dominated by interstitial permeability (k). The close agreement between first- and total-order indices indicates weak parameter interactions and an approximately additive response structure, supporting robust parameter identifiability and enabling reduced-order modeling.

A comparison between surrogate modeling strategies further highlights that MF-GP significantly outperforms neural networks (NNs) in data-scarce regimes typical of computational biomechanics. While NNs are flexible function approximators  (Linka et al. 2022), they exhibit reduced predictive accuracy and less reliable uncertainty estimates when trained on sparse HF datasets (Willard et al. 2022; Kong et al. 2023). In contrast, the MF-GP surrogate leverages correlations between HF and LF models to improve predictive accuracy and provide calibrated uncertainty estimates.

Across physiologically relevant regimes, MF-GP predictions of ${ C}_{rs}$ and ${ R}_{rs}$ show minimal bias, strong agreement with HF simulations, and good Bland–Altman consistency without evidence of systematic proportional bias. These results indicate that the surrogate captures the dominant behavior of the underlying poroelastic system within the explored parameter space. Agreement with literature ranges further supports physiological consistency (Herrmann et al. 2020), although it does not constitute full experimental validation.

From a computational perspective, the MF-GP surrogate provides substantial efficiency gains, reducing simulation time from hours for HF FE models to milliseconds for surrogate inference. This enables large-scale uncertainty propagation and sensitivity analyses that would otherwise be computationally prohibitive.

A limitation of the present study is that parameter identification is based solely on global scalar metrics, namely respiratory compliance (${ C}_{rs}$) and resistance (${ R}_{rs}$), which do not explicitly capture regional heterogeneities in lung mechanics (Herrmann et al. 2022). While these quantities are widely used in clinical and experimental practice and provide a practical basis for model calibration, they inherently average out spatial variations in tissue deformation and ventilation. It is important to note, however, that the underlying HF poroelastic FE model is fully spatially resolved and capable of representing regional distributions of stress, strain, and pressure within the lung parenchyma. The present focus on global observables was motivated by the need for computational efficiency and direct comparability with commonly available ventilator measurements. Nevertheless, incorporating spatially resolved data, such as imaging-derived ventilation fields or regional strain measurements, would enable spatially informed parameter estimation and improve the model’s ability to capture heterogeneous lung behavior. This represents an important direction for future work and would further enhance the physiological fidelity and clinical relevance of the proposed framework.

From a translational perspective, this work addresses a gap in surrogate-assisted poroelastic modeling for porcine lungs, which remain the primary preclinical model for ventilation and lung injury studies. However, further validation across broader datasets and ventilation conditions will be required to establish clinical or preclinical deployment.

## Conclusion

In summary, this study presents a multi-fidelity poroelastic finite-element and machine learning framework for efficient and uncertainty-aware modeling of porcine lung mechanics. The MF-GP surrogate enables accurate prediction of respiratory compliance and resistance with low error and substantial computational acceleration. Beyond predictive performance, the framework provides new insight into the structure of the inverse problem, demonstrating that a small subset of parameters governs system behavior and can be reliably identified from global measurements. This supports reduced-order modeling and improves the interpretability of lung mechanics. Overall, the proposed approach establishes a scalable and physiologically consistent pathway for parameter estimation, uncertainty quantification, and predictive modeling, with potential applications in personalized ventilation strategies, experimental design, and preclinical respiratory research.

## Data Availability

The data that support the findings of this study are available on https://github.com/cmchao2005/multi-fidelity-poroelastic-FE-and-ML-framework-for-lung.
